# Synthesis and Characterization of Ruthenium-Paraphenylene-Cyclopentadienyl Full-Sandwich Complexes: Cytotoxic Activity against A549 Lung Cancer Cell Line and DNA Binding Properties

**DOI:** 10.3390/molecules29010017

**Published:** 2023-12-19

**Authors:** Evangelia Sifnaiou, Theodoros Tsolis, Konstantinos Ypsilantis, Eugenia Roupakia, Evangelos Kolettas, John C. Plakatouras, Achilleas Garoufis

**Affiliations:** 1 Laboratory of Inorganic Chemistry, Department of Chemistry, University of Ioannina, 45110 Ioannina, Greece; e.sifnaiou@uoi.gr (E.S.); t.tsolis@uoi.gr (T.T.); k_ipsilantis@yahoo.gr (K.Y.); iplakatu@uoi.gr (J.C.P.); 2 Laboratory of Biology, School of Medicine, Faculty of Health Sciences, University of Ioannina, 45110 Ioannina, Greece; ev.roupakia@uoi.gr (E.R.); ekoletas@uoi.gr (E.K.); 3 Institute of Biomedical Research, Foundation for Research and Technology, 45110 Ioannina, Greece; 4 Institute of Materials Science and Computing, University Research Centre of Ioannina (URCI), 45110 Ioannina, Greece

**Keywords:** ruthenium, paraphenylene, cyclopentadienyl, A549 lung cancer, DNA binding

## Abstract

Novel full-sandwich (*η*^5^-Cp)-Ru-paraphenylene complexes with the general formula [(*η*^5^-Cp)_n_Ru(*η*^6^-L)](PF_6_)_n_ where n = 1–3 and L = biphenyl, *p*-terphenyl and *p*-quaterphenyl, were synthesized and characterized by means of spectroscopic and analytical techniques. The structures of the complexes [(*η*^5^-Cp)Ru(*η*^6^-biphenyl)](PF_6_) (**1**), [(*η*^5^-Cp)Ru(*η*^6^-terphenyl)](PF_6_) (**3**) and [(*η*^5^-Cp)_2_Ru(*η*^6^-terphenyl)](PF_6_)_2_ (**4**) was determined by X-ray single crystal methods. The interaction of the complexes [(*η*^5^-Cp)Ru(*η*^6^-quaterphenyl)]Cl, (**6**)Cl, and [(*η*^5^-Cp)_2_Ru(*η*^6^-quaterphenyl)]Cl_2_, (**7**)Cl_2_, with the DNA duplex d(5′-CGCGAATTCGCG-3′)_2_ was studied using NMR techniques. The results showed that both complexes interacted non-specifically with both the minor and major grooves of the helix. Specifically, (**6**)Cl exhibited partial binding through intercalation between the T7 and T8 bases of the sequence without disrupting the C–G and A–T hydrogen bonds. Fluorometric determination of the complexes’ binding constants revealed a significant influence of the number of connected phenyl rings in the paraphenylene ligand (L) on the binding affinity of their complexes with the d(5′-CGCGAATTCGCG-3′)_2_. The complexes (**6**)Cl and (**7**)Cl_2_ were found to be highly cytotoxic against the A549 lung cancer cell line, with complex (**6**) being more effective than (**7**) (IC_50_ for (**6**)Cl: 17.45 ± 2.1 μΜ, IC_50_ for (**7**)Cl_2_: 65.83 ± 1.8 μΜ) and with a selectivity index (SI) (SI for (**6**)Cl: 1.1 and SI for (**7**)Cl_2_: 4.8).

## 1. Introduction

Since the discovery of cisplatin [1], a plethora of metal ion compounds have exhibited notable cytotoxic activity. For the vast majority of these compounds, their action mechanism is associated with the coordination of the metal center to biological targets, such as proteins and DNA. This process bears a resemblance to the mechanism of alkylating chemotherapeutics [2]. However, there exists a less common category of coordination compounds that also exhibit cytotoxic properties. In this category, the ligands surrounding the metal’s coordination sphere are kinetically inert, and thus, their action mechanism differs entirely from the previous category. They cannot replace any of their ligands and, therefore, lack a coordination site. Typically, they non-coordinatively bind to proteins and/or DNA, inducing structural modifications that affect their functionality, akin to the behavior of some anticancer antibiotics, such as actinomycin D, bleomycin, daunomycin, etc. [3]. However, their efficacy depends on their ability to penetrate the cell membrane and their specificity for cancer cells. The latter is a crucial characteristic of ruthenium compounds, which have the ability to selectively accumulate in cancer cells through transferrin receptors [4], ranking them among the most promising agents for cancer chemotherapy. Most of them act by forming coordination bonds with DNA or with other biomolecules, inhibiting cellular proliferation [5]. However, many ruthenium complexes, particularly those with oligopyridine or polypyridine ligands, bind to DNA non-coordinatively, inducing structural alterations that can potentially trigger programmed cell death [5].

Current interest is directed toward full-sandwich cationic ruthenium compounds with arenes due to their notable cytotoxic properties, which can be partially attributed to their balance hydrophilic–lipophilic nature, high cellular uptake, and distribution [6]. Moreover, the high stability of these complexes in aqueous media, attributed to the absence of any potential good leaving group, suggests their capability to interact with biomolecules in a non-coordinative manner, exhibiting promising cytotoxic potential, as evidenced by several reports, reviewed below: Full-sandwich ruthenium complexes containing *η*^6^-coordinated *p*-cymene and mono- and di-selenoquinones, have been synthesized and characterized. The complexes display moderate cytotoxicity against A2780 and A278R cancer cell lines, depending on their structure with IC_50_ in the range of 19 to 240 μΜ [7]. The cytotoxic activity of the complex [(*η*^5^-Cp*)Ru(*η*^6^-C_6_H_5_CO_2_H)]PF_6_, along with its various esters and amides, was assessed in vitro against the MCF7, MDA-MD-231, and MM96L cancer cell lines. The esters of the complex demonstrated a higher level of activity compared to the amides and carboxylic acid derivatives, probably due to their higher lipophilicity, leading to an increased cellular uptake [8]. A series of novel full-sandwich complexes with the general formula [(*η*^5^-Cp*)Ru(*η*^6^-arene)]^+^ was examined for their cytotoxicity against various human cancer cell lines, as well as normal human fibroblasts. The results showed that their anticancer activity varies with changes in the arene ligand and the anionic counterion [9]. In vitro cytotoxicity investigations of the [(*η*^5^-Cp*)Ru(PhNHCO_2_R)]BPh_4_ (R = Me, Et, and *n*-Pr) have revealed that they are potent growth inhibitors for diverse cancer cell lines, while they exhibited significantly lower levels of toxicity towards a normal human fibroblast cell line [10]. A similar moderate selective cytotoxicity at low micromolar concentrations has been observed for the full-sandwich complexes Cp*Ru(benzosulfonamides) screened at various cancer cell lines. These complexes demonstrated a noteworthy ability to hinder various human carbonic anhydrase isoenzymes I and II, mitochondrial isoenzymes VA and VB, along with the cancer-linked isoenzyme IX [11]. Cationic full-sandwich Cp*-ruthenium complexes with *η*^6^-benzyl glucose of the formula [(*η*^5^-Cp*)Ru(*η*^6^-benzylglucose)]Cl, [benzylglucose = peracetylated benzyl β-d-glucopyranoside and benzyl β-d-glucopyranoside] exhibited antimigration and anti-invasive activity against MDA-MB-231 and cisplatin-resistant SK-OV-3 cancer cells lines. These complexes were found to be non-toxic against non-cancerous human kidney cells (HEK293) [12]. The interactions of full-sandwich complexes [(*η*^5^-Cp*)Ru(*η*^6^-arene)] with the plasmid DNA pBR322 have been studied by atomic force microscopy. The results demonstrated a strong non-coordinative interaction depending on the nature of the arene. However, it should be noted that the complexes exhibited low cytotoxicity against human leukemia cancer cell line (HL-60) [13]. Conjugating ruthenocene in a peptide nucleic acid (PNA) results in low cytotoxicity against several cancer cell lines despite its significantly high cellular uptake. The latter, together with its low toxicity and high stability, make it a promising agent for bioanalytical applications [14]. A series of lipophilic, cationic (*η*^5^-Cp*) full-sandwich complexes were assessed for their cytotoxicity against various cancer cell lines. The results indicate that they exhibited significant cytotoxicity with IC_50_ values ranging in the micromolar scale [15]. The cytotoxic activity against two multiple myeloma cell lines of novel full-sandwich quinoline complexes, *η*^5^-Cp-Ru-*η*^6^-quinoline, and their stability in vitro and in cell culture was investigated. Despite that, the complexes showed poor cellular proteasome inhibition and demonstrated good cytotoxicity with IC_50_ of a few μM [16]. Neutral ruthenocenyl complexes, including substituted Cp ligands, show weaker cytotoxic activity in comparison to similar cationic counterparts. This suggests that the presence of the positive charge in complexes [(*η*^5^-cyclopentadienyl)Ru(*η*^6^-arene)]^+^ complexes enhances their biological activity [17]. Also, the neutral dimethylated acid anhydride of the formula [Ru(*η*^5^-C_5_H_5_) (*η*^5^-C_5_H_4_CO)]_2_O was assessed for its antiproliferative ability against various cancer cell lines, along with human fibroblasts. The results suggest that these organoruthenium metallocenes exhibit moderate to weak cytotoxicity against cancer cells [18]. The neutral complexes (*η*^6^-arene)ruthenacarborane, (arene = *p*-cymene, biphenyl, and 1-Me-4-COOEt-C_6_H_4_) were found to exhibit moderate activity against HCT116 and MCF-7 cancer cell lines, while they are non-toxic against normal cells [19].

**Scheme 1:**
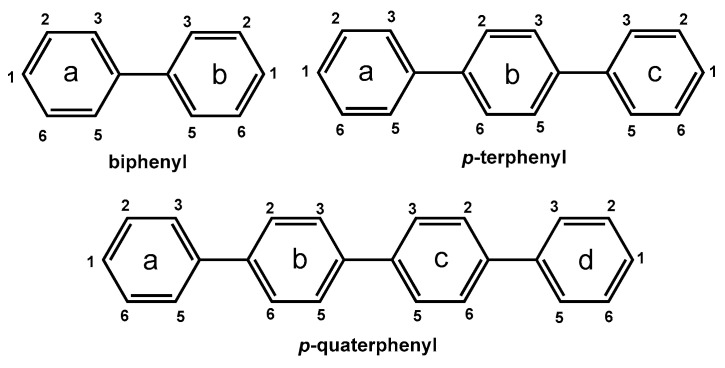
Structures and numbering of ligands used in this study.

In order to combine, (a) the capability of kinetically inert complexes containing ligands capable of binding to DNA, (b) the tendency of ruthenium complexes to accumulate in cancer cells, and (c) the balance between the hydrophilic–lipophilic nature of ruthenium- arenes complexes and their high cellular uptake and distribution, we herewith report on the synthesis and characterization of full-sandwich (*η*^5^-Cp)-Ru-paraphenylene complexes with the general formula [(*η*^5^-Cp)_n_Ru(*η*^6^-L)](PF_6_)_n_ where n = 1–3 and L = biphenyl, *p*-terphenyl and *p*-quaterphenyl (Scheme 1). Moreover, we studied the DNA binding properties of selected complexes using as B-DNA model the synthetic oligonucleotide duplex d(5′-CGCGAATTCGCG-3′)_2_ using NMR techniques [20] and fluorescence titrations [21] as well as their cytotoxic activity against A549 lung cancer cells. This specific cell line was chosen as a model [22] to investigate the cytotoxic activity of the selected ruthenium compound, given the high incidence and mortality rate of lung cancer [23], emphasizing the need for discovering novel chemotherapeutic agents [24].

## 2. Results and Discussion

### 2.1. Synthesis and Characterization of the Complexes *(**1**)–(**8**)*

#### 2.1.1. Synthesis

The complexes (**1**)–(**8**) were synthesized by reacting *p*-paraphenylene (biphenyl, *p*-terphenyl, *p*-quaterphenyl) with the compound [(*η^5^*-C_5_H_5_)Ru(CH_3_CN)_3_](PF_6_) in dry and degassed dichloromethane or acetone.

The complexes with 1:1 ratio L: {RuCp}, (**1**) and (**3**), were synthesized using equimolar amounts of ligands and [(*η*^5^-C_5_H_5_)Ru(CH_3_CN)_3_]PF_6_, were single charged cationic complexes and isolated as PF_6_ salts. Unreacted materials and reaction byproducts were removed from the crude solid by washing with H_2_O and CH_2_Cl_2_, but some amount of the main products dissolved as well in this process. In the case of (**6**), [(*η*^5^-C_5_H_5_)Ru(*η*^6^*-p*-quaterphenyl)]PF_6_, the 1:1 molar ratio led very easily to a mixture of 1:1 and 1:2, most likely due to the distant coordination sites of the four phenyl rings, which the {RuCp} unit associated. Therefore, the synthesis of (**6**) proceeded using a three-fold excess of *p*-quaterphenyl, which was subsequently removed by washing the crude product with CH_2_Cl_2_. The final complex is taken as the soluble part of the crude product in acetone, where the *p*-quaterphenyl is insoluble.

**Scheme 2:**
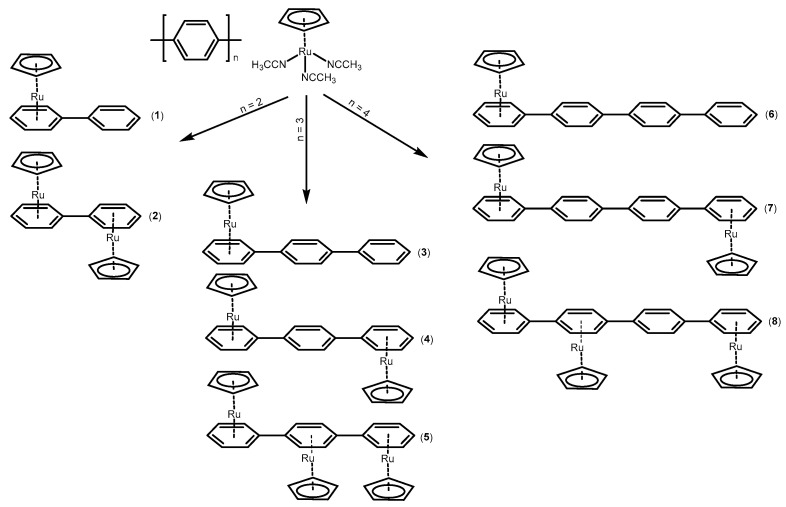
Synthetic procedures for the complexes (**1**)–(**8**).

The synthesis of the fully coordinated biphenyl by two {RuCp} units, (**2**), was easily performed by using a slight excess of 2.5 eq. of [(*η*^5^-C_5_H_5_)Ru(CH_3_CN)_3_](PF_6_) in the reaction mixture. Similarly, the complex (**5**) was synthesized. However, the synthesis of the 1:2, L: ({RuCp}, complexes (**4**) and (**7**) were achieved through successive in situ reactions. Initially, the 1:1 complex was formed, as described in the cases of (**3**) and (**6**), and without isolating them, additional amounts of [(*η*^5^-C_5_H_5_)Ru(CH_3_CN)_3_](PF_6_) were added. In the context of the successive reactions, the synthesis of complex (**8**) was achieved. Attempts to synthesize the fully coordinated *p*-quaterphenyl by four {RuCp} units failed, even when employing a 10-fold excess of [(*η*^5^-C_5_H_5_)Ru(CH_3_CN)_3_](PF_6_). Also, we did not detect the four-coordinated complex in the ^1^H NMR or ESI-MS spectra of the reaction mixture, indicating that steric or electronic factors inhibit the formation of such a complex. We notified that the synthesis of the similar complex [(*η*^5^-Cp*)_4_Ru(*η*^6^-terphenyl)](OTf)_4_ (Cp* = *η*^5^-C_5_(CH_3_)_5_, OTf = CF_3_SO_3_) has been reported [25]. The synthetic procedures are summarized in Scheme 2.

#### 2.1.2. Solution Characterization

The complexes were characterized by NMR techniques, high-resolution ESI-MS, and single-crystal X-ray diffraction methods.

The ^1^H NMR spectrum of biphenyl is simple due to its high symmetry, showing only three signals assigned to H_1a/1b_, H_2a6a/2b6b_, and H_3a5a/3b5b_. Similarly, complex (**2**) also displays high symmetry, exhibiting three signals attributed to biphenyl protons, along with a five-proton singlet assigned to Cp moiety. However, the signals from biphenyl shifted approximately 1 ppm upfield, in comparison with the free ligand, reflecting the ruthenium’s contribution to the arene electron density. This effect is more pronounced in the case of (**1**), where six signals of biphenyl appear in the spectrum. Three of them shifted by 1 ppm upfield, while the other three, corresponding to the free ring, remained almost unchanged. In a manner similar to biphenyl, the spectrum of *p*-terphenyl shows four signals, as does the spectrum of (**5**) alongside the signals from the Cp protons. The *p*-terphenyl protons shifted upfield at about 0.8 ppm, as expected. However, the three Cp moieties appear to exhibit non-equivalence. A 10-proton singlet at 5.62 ppm and a 5-proton singlet at 5.72 ppm represents the two different coordination environments of the {RuCp} moieties. This difference can be attributed to the positioning of the three {RuCp} units, with two {RuCp} being coordinated from one side of *p*-terphenyl and the other one from the opposite side (Figure S8b). Complex (**4**) also exhibits a high symmetry, and in its ^1^H NMR spectrum, only one set of upfield shifted signals was observed. The proton signals of the middle ring remain almost unchanged, indicating that the {RuCp} units coordinated to the end rings (Figure S8c). In the spectrum of (**3**), the two end rings of *p*-terphenyl appear to exhibit non-equivalence as well. One set of signals is observed upfield, at about 1 ppm, and can assigned to the ring, which is *η*^6^-coordinated with the {RuCp} unit. The other set is almost identical to the signals of the free ligand. Additionally, the signal of the middle ring protons remains unchanged. These observations support the conclusion that one of the end rings is associated with the {RuCp} unit (Figure S8d). It appears that in the 1:1 complex with *p*-terphenyl, the *η*^6^-coordination of the {RuCp} unit in the middle ring is not favored for electronic reasons. Nevertheless, a *p*-terphenyl complex with the coordinated unit of {RuCp*} in the middle ring has been reported. Its synthesis was achieved stepwise, starting with 1,4-dibromobenzene associated with the {RuCp*} unit. Then, the two other phenyl rings were added on either side by a Suzuki coupling reaction [26].

**Figure 1:**
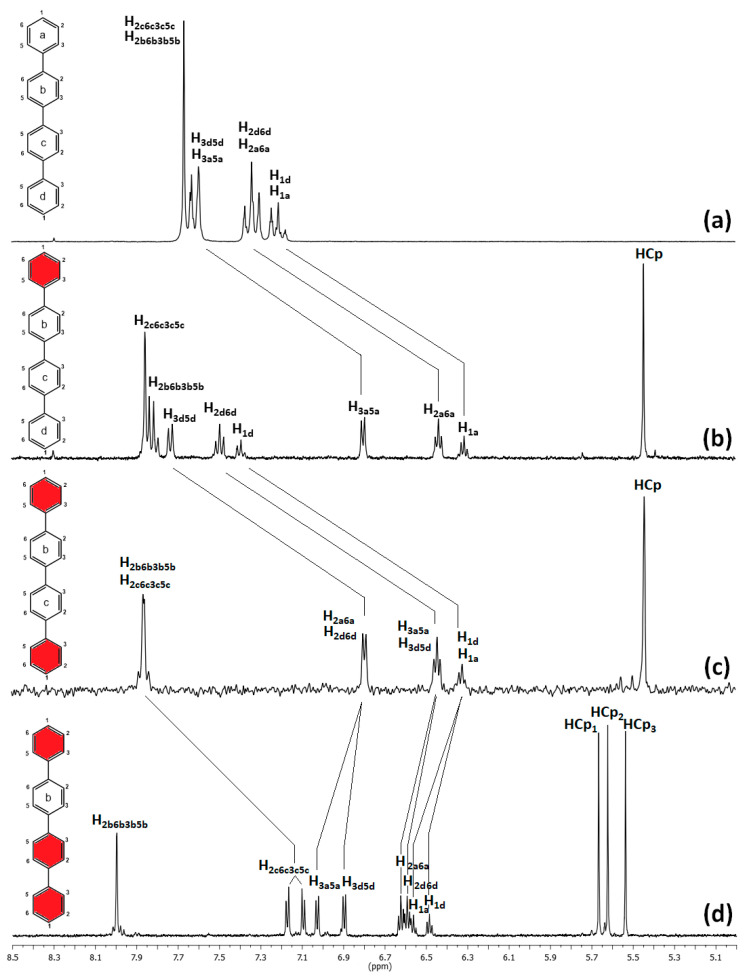
^1^H NMR spectra (dmso-*d*_6_, 298 K) of the (**a**) free *p*-quaterphenyl, and (**b**–**d**) the complexes (**6**)–(**8**), with structure numbering and assignments. The phenyl rings in red indicate that they are *η^6^*-coordinated with {RuCp}.

In the ^1^H NMR spectrum of the *p*-quaterphenyl, the proton signals of the two terminal phenyl rings, (a) and (d), appear to be identical, as well as the signals of the middle rings, (b) and (c). Upon coordination of one {RuCp} unit in complex (**6**), the proton signals of one terminal phenyl ring shift upfield by 1 ppm, indicating that the Ru is *η*^6^-coordinated to the (a) phenyl ring of *p*-quaterphenyl (Figure 1b). The proton signals of the other phenyl rings shifted slightly downfield, and the proton signals of ring (b) are no longer identical to those of ring (c), as the symmetry is disrupted by the neighboring *η*^6^-coordinated ring (a). Similarly, in the spectrum of (**7**), all the signals of rings (a) and (b) shifted upfield, indicating that both rings are coordinated with an {RuCp} unit. Also, the middle rings (b) and (c) appear to be identical, confirming the high symmetry of (**7**) (Figure 1c). In the spectrum of (**8**), three different Cp five-proton signals appear, showing that there are three non-equivalent {RuCp} units on the *p*-quaterphenyl molecule. For this condition, only one arrangement is possible, in which the phenyl rings (a), (b), and (d) are coordinated. Indeed, upfield shifts for the proton signals of these rings occur, while the signals of (b) remain almost unchanged. However, the proton signals of the end rings are not equal, probably due to the presence of a neighboring {RuCp} unit in only one ring (Figure 1d).

The HR-ESI-MS spectra of the prepared complexes (**4**)–(**8**) show simple, double, or triple-charged cations in accordance with their proposed formulae (Figures S1–S5).

#### 2.1.3. Crystal Structure of the Complexes (**1**), (**3**) and (**4**)

**Figure 2:**
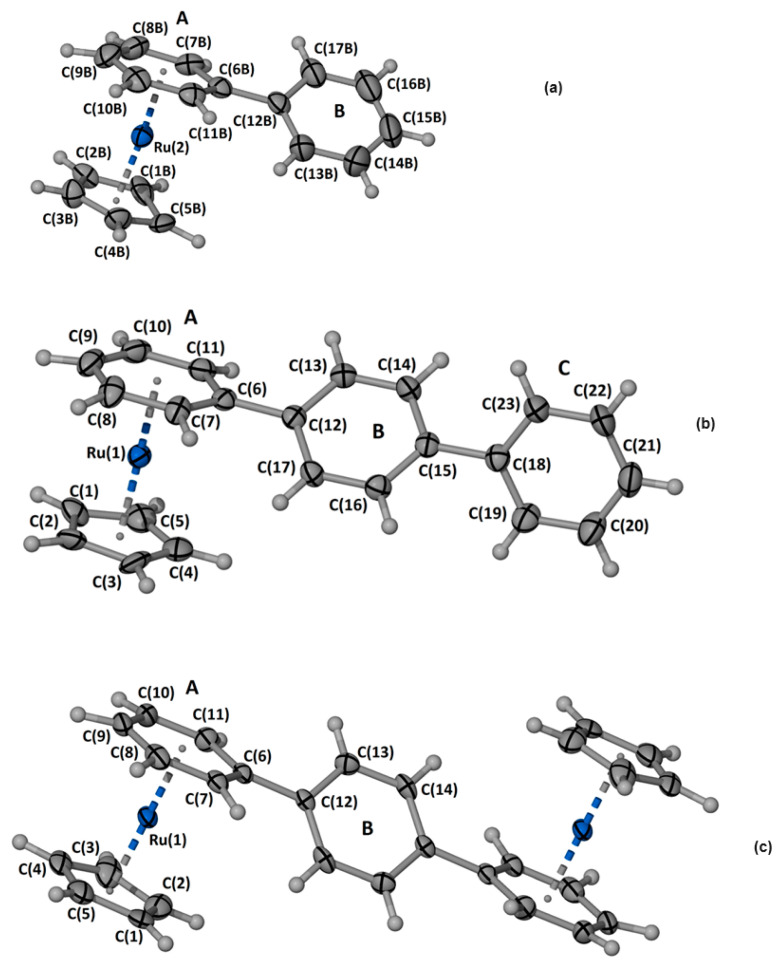
Thermal ellipsoid plots (40% probability level) of the cations in the structurally characterized compounds, showing the labeling scheme. Designation of the C_6_ rings of the paraphenylene ligands is also included. Only one part of the disordered cyclopentadienyl moieties is shown for clarity. (**a**) Compound (**1**), only one of the two cations present in the asymmetric unit is shown; (**b**) Compound (**3**); (**c**) Compound (**4**), symmetry operation to generate equivalent atoms: −x + 2, −y + 1, −z + 1.

Crystals, suitable for X-ray diffraction studies, were grown by vapor diffusion of diethyl ether in a dichloromethane solution of the corresponding compound. The structures of the cations are shown in Figure 2, while selected structural characteristics for them are presented in Table 1.

All compounds crystallize in centrosymmetric space groups with mixed-sandwich Ru cations and ${PF}_{6}^{-}$ anions in the unit cell. The cyclopentadienyl ligands are disordered over two positions in all compounds.

(**1**), formulated as [(*η^5^*-C_5_H_5_)Ru(*η*^6^-biphenyl)](PF_6_), is crystallized in the monoclinic space group *P*$\bar{1}$ with two ionic pairs with slightly different geometrical characteristics in the asymmetric unit. (**3**) is monoclinic in space group *C*2/*c*. Its asymmetric unit contains a cation formulated as [(*η*^5^-C_5_H_5_)Ru(*η*^6^-*p*-terphenyl)]^+^ and two halves of PF_6_^−^ with the phosphorus atoms lying on a rotoinversion axis. (**4**) is also monoclinic with space group *P*2_1_/*n*, formulated as [(*η*^5^-C_5_H_5_)_2_Ru_2_(*η*^6^-*p*-terphenyl)](PF_6_)_2_, with the asymmetric unit containing one half of the cation and one PF_6_^−^ balancing the charge.

The mean C–C and M–C bond lengths of the coordinated ligands fall within the range of other (*η*^6^-benzene)(*η*^5^-cyclopentadienyl)ruthenium(II) cations [13,27,28]. A close examination of the bond distances and angles between the two coordinated rings (Table 1) is indicative of a C_5_–Ru–C_6_ coordination mode for all compounds; practically, the Centroid C_5_–Ru–Centroid C_6_ angles are very close to 180°. In all three complexes, the ruthenium atom is not centered between the cyclopentadienyl and the benzene rings but is shifted toward the benzene ring, underlying the fact that the larger ring system allows deeper immersion of the ruthenium atom in the coordination sphere.

A common characteristic of the three cations is the twisted orientation of non-coordinated C_6_ rings of the biphenyl and para-terphenyl ligands with respect to the least square planes of the coordinated C_6_ rings.

It is likely that this orientation of the aromatic ligand rings is adopted to relieve the steric repulsion between the hydrogen atoms bonded on the ortho positions of the ring connecting carbon atoms.

It is worth noting that there are no significant π–π stacking interactions in all three structurally characterized compounds. However, there is a plethora of non-conventional C–H ⋅⋅⋅ F hydrogen bonds that stabilize the arrangement in the crystal lattice.

### 2.2. Biological Studies

To investigate the biological properties of complexes (**1**)–(**8**), we transformed them into corresponding water-soluble analogs. This was achieved by converting their [PF_6_]^−^ salts to [Cl]^−^, as described previously [21,29]. The HR-ESI mass spectra of the chloride salts of the complexes were identical to that of their hexafluorophosphates.

#### 2.2.1. Fluorescence Quenching Studies of the d(5′-CGCGAATTCGCG-3′)_2_-Ethidium Bromide Adduct, with the Complexes (**1**)–(**8**)

Fluorescence spectroscopy is a sensitive and efficient method used to investigate the binding modes of small molecules with DNA [30]. Ethidium bromide (EtBr), a cationic dye, is well-known for its strong affinity for DNA, primarily through intercalation, which significantly enhances DNA fluorescence [31,32]. The displacement of EtBr from a DNA–EtBr adduct by a DNA binder serves as evidence that the latter also intercalates, resulting in a significant reduction in emission intensity. The extent of the quenching directly reflects the magnitude of the competition constant. However, EtBr can also bind to DNA through the helix minor groove as well as by electrostatic interactions due to its cationic nature [33]. In the case of displacement from the minor groove, the magnitude of DNA–EtBr intensity decrease is smaller [34]. Molecular simulation studies of the interactions between the Drew–Dickerson dodecamer d(5′-CGCGAATTCGCG-3′)_2_ and the EtBr reveal that mainly stacked on or intercalated between the terminal base pairs CG of the duplex, with little to no interaction with the inner base pairs AT [35].

**Figure 3:**
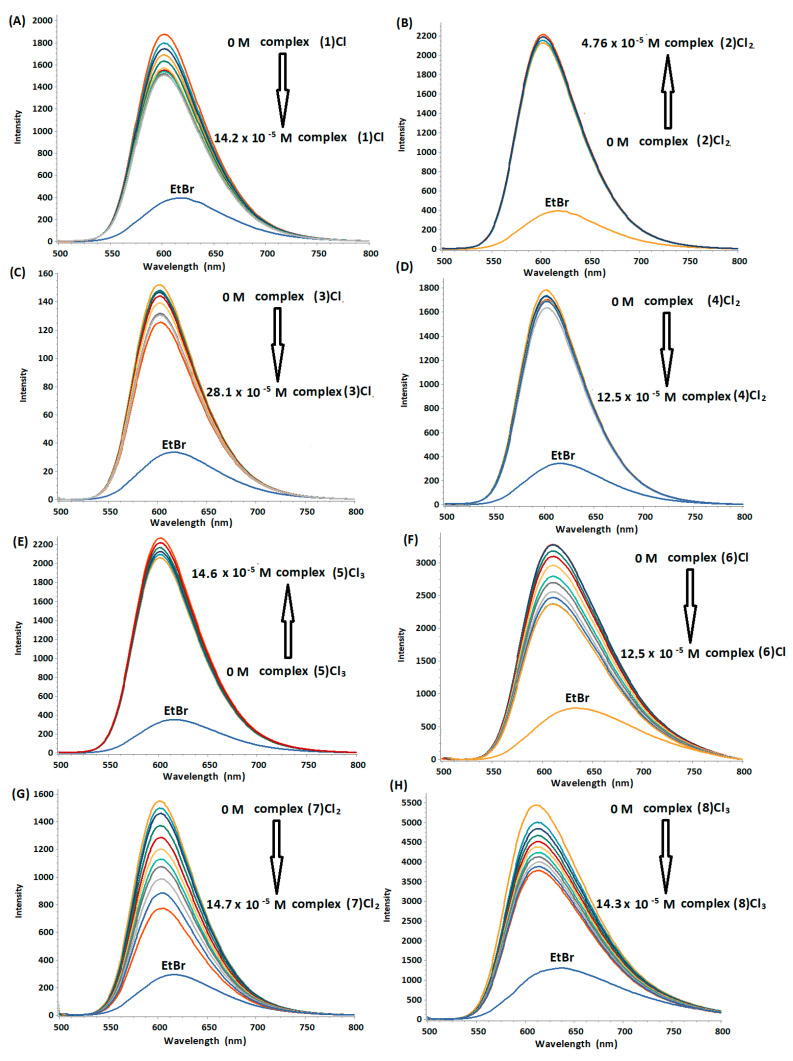
Fluorescence emission spectra of DNA–EtBr titrated with chloride salts of the complexes (**1**)–(**8**) at 298 K. [DNA] = 20 μM, [EtBr] = 5.2 μM, and [complex] = 0 to 20.10 μΜ. (**A**) (**1**)Cl, (**B**) (**2**)Cl_2_, (**C**) (**3**)Cl, (**D**) (**4**)Cl_2_, (**E**) (**5**)Cl_3_, (**F**) (**6**)Cl, (**G**) (**7**)Cl_2_, and (**H**) (**8**)Cl_3_.

Initially, we prepared a solution d(5′-CGCGAATTCGCG-3′)_2_ in buffer phosphates (100 mM, pH 7.0) to which we added an amount of EtBr until the emission intensity reached a point where any further addition has a negligible impact. Samples of the above stock solution were titrated with the chloride salts of the complexes (**1**)–(**8**). In the cases of (**1**)Cl, (**3**)Cl, (**4**)Cl_2_, (**6**)Cl, (**7**)Cl_2_, and (**8**)Cl_3_, the emission intensity of the DNA–EtBr adducts decreased to varying degrees as the concentration of the complexes increased, while the wavelength of the maximum emission remained almost unchanged (Figure 3). These results indicate the displacement of the EtBr from the DNA–EtBr adduct due to the binding of the complexes either through intercalation or through binding to the helix minor groove [36,37,38]. This is precisely reflected in the competition Stern–Volmer quenching constant, in which the values were determined from the slopes of the F/F_o_ = f([Q]) plots (Figure S6). The fluorescence quenching of the DNA–EtBr adduct was in good agreement with the Stern–Volmer linear equation (R > 0.98), with quenching percentages of the complexes ranging from 11% to 66%. On the other hand, the intensity of DNA–EtBr increased upon increasing the concentration of complexes (**2**)Cl_2_ and (**5**)Cl_3_.

The binding constants (K_b_) and the number of binding sites (n) were estimated using fluorescence titration data and calculated through the double logarithmic plot log [F_0_ - F/F] versus log[Q] (Figure S7). The values of the binding constant were in the order of magnitude of 10^3^ M^−1^, indicating moderate binding of the complexes with the number of binding sites practically being one [39]. The results are summarized in Table 2.

The above results suggest that among the complexes studied, (**6**)Cl, (**7**)Cl_2_, and (**8**)Cl_3_ exhibit the highest effectiveness in displacing EtBr, with an overall tendency being (**7**) > (**6**) > (**8**) > (**3**) > (**1**) > (**4**). Additionally, the binding constants of *p*-quaterphenyl complexes were greater than those of corresponding biphenyl and *p*-terphenyl complexes, showing that the number of the attached phenyl rings of the paraphenylene significantly affects the binding strength of the complexes to DNA. Moreover, the coordination degree of the {RuCp} unit to the paraphenylene ligand also plays a crucial role in the interaction with the DNA. Mono-coordinated paraphenylenes strongly interact with the DNA, whereas fully coordinated complexes with the {RuCp} unit, such as (**2**)Cl_2_ and (**5**)Cl_2_, appeared to interact electrostatically. This interaction slightly increases the emission intensity of the DNA–EtBr adduct. However, neither the Ksv nor Kb constants, along with the displacement extent of the EtBr, reached significant levels, suggesting that the studied complexes likely act as groove binders.

#### 2.2.2. Cytotoxic Activity

The complexes (**6**)Cl and (**7**)Cl_2_ were chosen for further studies due to their observed stronger interactions with DNA compared to the others.

**Figure 4:**
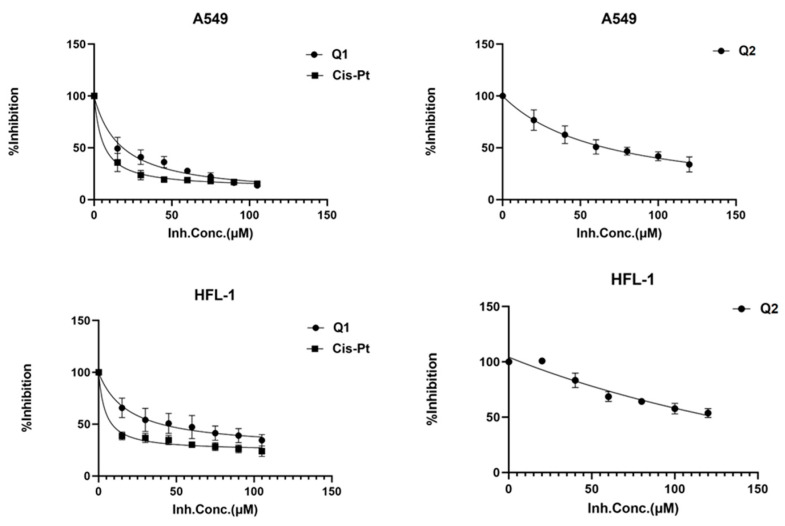
Plots of the % cell viability vs. the concentrations of (**6**)Cl, (**7**)Cl_2_, and cis-Pt against the human lung cancer cell line A549 and the human fetal lung fibroblasts HFL-1. Results represent the average of three independent experiments.

The in vitro cytotoxic activities of complexes (**6**)Cl and (**7**)Cl_2_ were assessed against human A549 cancer cells and normal human fibroblasts HFL-1, titrated with increasing concentrations of these complexes over a 48 h period. For comparison, the IC_50_ value of cisplatin was also calculated (Figure 4 and Table 3). In addition to the IC_50_ value, the cytotoxic activity was also assessed in terms of selectivity index (SI), where a higher SI suggests greater selectivity for cancer cells and lower toxicity to normal cells. Among the tested compounds, cisplatin showed the highest cytotoxicity with an IC_50_ value of 5.49 ± 2.1 μM and a selectivity index SI = 0.8. Significantly, complex (**6**)Cl demonstrated notable efficacy, displaying an IC_50_ value of 17.45 ± 2.1 μM and a higher selectivity index than cisplatin. Similarly, despite the relatively higher IC_50_ of 65.83 ± 1.8 μM, it shows a selectivity index five times better than cisplatin.

#### 2.2.3. NMR Studies of the Interactions of the Complexes (**6**)Cl and (**7**)Cl_2_ with the d(5′-CGCGAATTCGCG-3′)_2_

The complexes (**6**)Cl and (**7**)Cl_2_ were chosen to undergo a detailed investigation of their binding mode with the DNA fragment d(5′-CGCGAATTCGCG-3′)_2_ using 1D ^1^H NMR and 2D NOESY spectroscopic techniques. The spectra of the DNA were recorded in H_2_O:D_2_O (9:1) at phosphate buffer solution (100 mM, pH = 7.0) (Figure S9). Under the same conditions, the spectra of the complexes (**6**)Cl and (**7**)Cl_2_ were recorded, as well (Figures S10 and S11). Subsequently, the DNA was titrated with the complexes at increasing molar ratio, and the mixture was incubated at 298 K for a period of 30 min, after which the spectra were recorded again (Figure S12 for complex (**6**) and Figures S15, S18 and S20 for complex (**7**)). The assignments of the proton signals were assisted by COSY and NOESY experiments (Figures S13 and S14 for complex (**6**) and Figures S16, S17, S19, S21 and S22 for complex (**7**)).

##### [(*η*^5^-C_5_H_5_)_2_Ru_2_(*η*^6^-*p*-quaterphenyl)]Cl_2_, (**7**)Cl_2_

**Figure 5:**
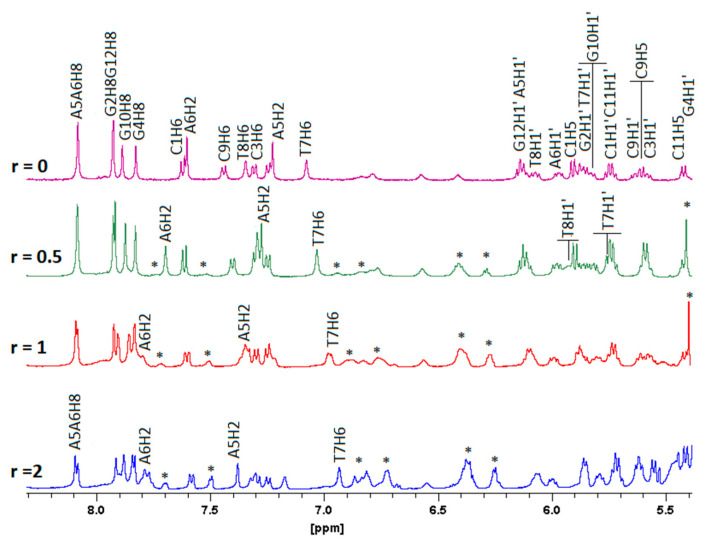
Stacked ^1^H NMR spectra of d(CGCGAATTCGCG)_2_ upon addition of complex (**7**)Cl_2_ at r = 0.5, r = 1, r = 2. The complex signals were denoted with an asterisk (*).

The ^1^HNMR spectrum of the complex (**7**)Cl_2_ in aqueous phosphate buffer closely resembles its [PF_6_]^−^ analog. Upon its addition to DNA at r = 0.5 ([complex]:[nucleotide]), the symmetry of the complex was reduced, and broad proton signals for each phenyl ring (a, b, c, d) appeared (Figure 5). As the broad signals were not easy to assign, the assistance of the COSY spectrum of the mixture was used. The two terminal rings, (a) and (d), exhibited chemical non-equivalence, showing downfield shifts in the range of 0.05 to 0.23 ppm, indicating a withdrawal of electron density, likely attributed to the involvement of the (b) and (c) phenyl rings in a bound interaction with the DNA. This electron density withdrawal is more pronounced for the adjacent rings to (b) and (c) protons, H_3a5a_ and H_3d5d_. This effect is dissimilar for the two rings. In contrast, the proton signals from the middle rings (b) and (c), showing significant upfield shifts (0.08–0.32 ppm), appeared as separate signals for H_2b6b_ and H_3b5b_ for each ring. This indicates that these rings are located in a region rich in electron density within the DNA helix. Notably, the Cp proton signals remained almost unchanged, indicating the absence of the {RuCp} unit of the binding process.

At r = 1, similar results were obtained, with slightly higher upfield shifts for the signals of the complex middle rings. However, the downfield shifts of the proton signals of the rings (a) and (d) decreased towards those of the free complex, suggesting a shift of the equilibrium towards the unbound state. This could be attributed to the DNA saturation at r = 0.5 or, alternatively, to a structural alteration of DNA due to the complex binding, resulting in changes in the environment of {RuCp} coordinated rings (a) and (d).

At r = 2, the excess of complex causes further shifts in the signals of rings (a) and (d) towards those of the free (**7**)Cl_2_, while the signals from the rings (b) and (c) remained almost intact. Additionally, new signals pop up from the protons of the rings (a) and (d), matching those of the free (**7**)Cl_2_. These results suggest that the complex primarily binds to DNA through the middle rings, with the coordinated by {RuCp} rings, (a) and (d), playing a minor role in the binding. Furthermore, it appears that one (**7**)Cl_2_ saturates the DNA helix from r = 0.5 (1:2, complex: strand). Table S4 and Figure S16 illustrate the above findings.

**Figure 6:**
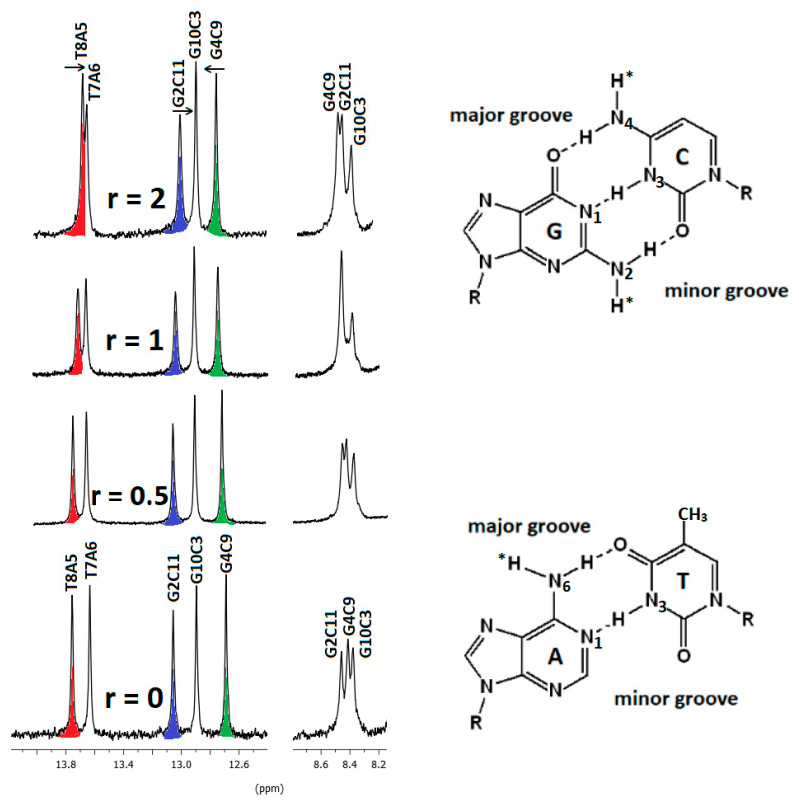
Part of the ^1^H NMR spectra of the d(5′-CGCGAATTCGCG-3′)_2_: free (r = 0), and upon addition of (**6**)Cl_2_ at r = 0.5, 1 and 2, showing the imino protons contributing the hydrogen bonds of G2N1**H**–C11N3, G10N1**H**–C3N3, G4N1**H**–C9N3, T8N3**H**–A5N1, and T7N3**H**–A6N1, as well as the amino protons G2N2**H**–C11O6/C11N4**H**–G2O6, G4N2**H**–C9O6/C9N4**H**–G4O6, G10N2**H**–C3O6/C3N4**H**–G10O6. Inset, a GC and an AT base pair show the positioning of the major and minor groove of a DNA helix.

Exchangeable imino and amino protons of the DNA bases are observable in H_2_O/D_2_O (9:1), and their chemical shifts provide evidence about the Watson–Crick (W.–C.) hydrogen bonds between the DNA strands (Figure 6, Table S5). The imino protons of G10, G4, and G12N1**H** form hydrogen bonds with the C3, C9, and C1N3, respectively, while the T8 and T7N3**H** are hydrogen bonding with the A5 and A6N1. The other hydrogen bonds between the base pairs are formed by one proton of the exocyclic amino group and the carbonyl group of the complementary base. The remaining non-hydrogen bonding proton in the exocyclic amino group symbolized as H* and in the ^1^H NMR spectra observed upfield (6.30 to 6.80 ppm) compared to the hydrogen-bonded one (8.30 to 8.50 ppm). At the ends of the sequence, G12N1**H** and C1N3 are either not hydrogen bonding, or their imino and amino protons were exchanged rapidly with the solvent, making them not observable [40].

Upon the addition of the complex to the DNA, the hydrogen bonding T8N3–**H**–A5N1 shifted upfield gradually as the ratio increased, reaching a value of −0.1 ppm at r = 2. The magnitude of this upfield shift suggests a possible elongation of the hydrogen bond or proximity in an electron-rich environment. Simultaneously, the T7N3–**H**–A6N1 hydrogen bond remained intact, indicating an absence of perturbation of the DNA helix. Within the GC base pairs of the sequence, the G10N1–**H**–C3N3 hydrogen bond remained unaffected. However, either the G10N2**H** or C3N4**H** hydrogen-bonded proton shifted slightly upfield. Additionally, the G4N1–**H**–C9N3 shifted downfield by 0.07 ppm at r = 2, whereas the G2N1–**H**–C11N3 shifted upfield by 0.05 ppm at r = 2. The above observations suggest that the (**7**)Cl_2_ binds to DNA through the grooves of the helix. However, it remains uncertain which specific groove facilitates this binding. Moreover, the presence of unbroken hydrogen bonds between the sequence strands, even at r = 2, dismisses any possibility of (**7**)Cl_2_ intercalation between the bases. The varied upfield and downfield shifts observed may be attributed to the binding impact on the helix’s geometry. The determination of which groove the complex associates with the DNA helix could be answered by inspecting the induced shifts by the (**7**)Cl_2_ of the non-exchangeable protons of the sequence (Table S6).

**Figure 7:**
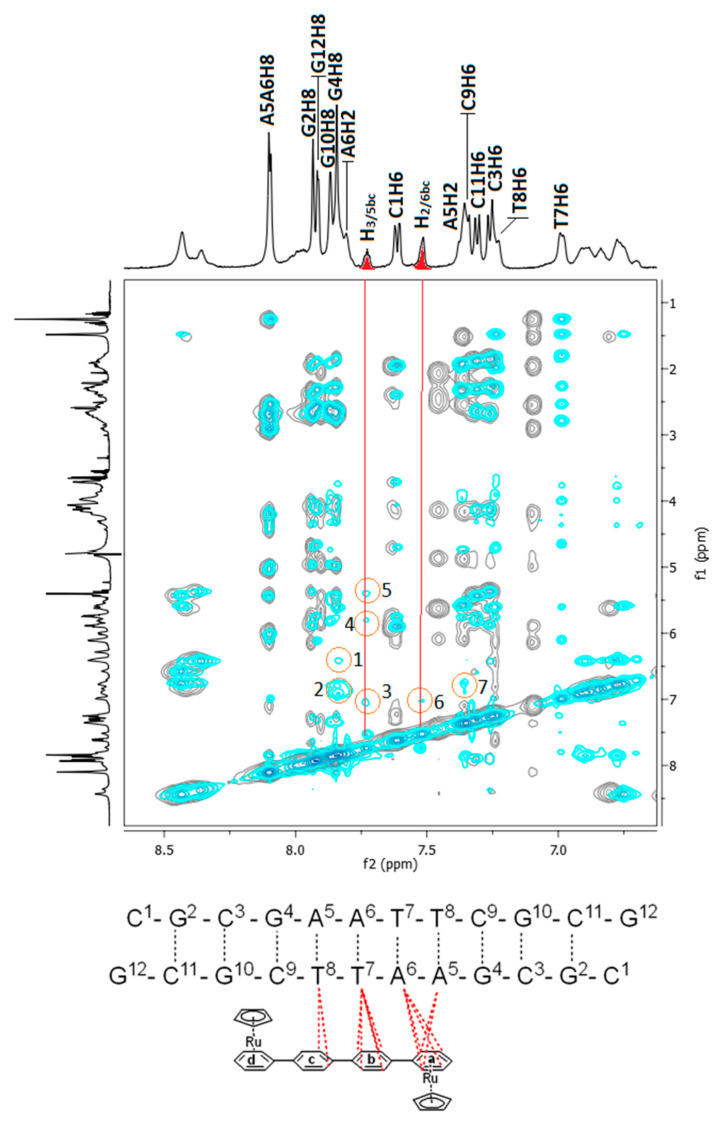
Superimposed NOESY spectra of the free d(5′-CGCGAATTCGCG-3′)_2_, gray scale, and its 1:1 mixture with (**7**)Cl_2_, light-blue scale (H_2_O:D_2_O, 9:1, 298 K, phosphates 100 mM, pH = 7.0 mixing time 350 ms). In red circles, new cross-peaks appeared only in the light-blue spectrum between (**7**)Cl_2_ protons and the d(5′-CGCGAATTCGCG-3′)_2_ protons. (1): A6H2→H_2a6a_, (2): A6H2→H_3aH5a_, (3): H_3b5b/3c5c_→T7H6, (4): H H_3b5b/3c5c_→T8H1′, (5): H H_3b5b/3c5c_→C9H1′ or T7H1′, (6): H_2b6b/2c6c_→T7H6, (7): A5H2→H_3a5a_.

Indeed, during the DNA titration with (**7**)Cl_2_ at r = 0.5, the signals corresponding to the aromatic protons of T8H6 and T7H6 shifted significantly upfield, as well as the sugar H1′ of T7, T8, and C9, indicating increased electron density surrounding these protons. In parallel, the H2 proton signals of A5 and A6 shifted downfield by +0.05 and +0.10 ppm, respectively, probably due to their interaction with the {RuCp} coordinated rings (a) and (d), which possess a partial positive charge. Upon increasing the ratio to r = 1, we observed significant upfield shifts of about −0.10 ppm for the aromatic protons of T8H6, T7H6, and C9H6000. Also, the observed upfield shifts at r = 0.5 nearly doubled in magnitude. Interestingly, the rest of the DNA proton signals exhibited only marginal shifts (Table S6). At r = 2, further upfield shifts were observed in neighboring DNA regions, including T7CH_3,_ the C9H5, and G10H8. Since the chemical shifts of the other DNA protons remain almost unchanged, it is assumed that (**7**)Cl_2_ binds along the sequence –A5A6T7T8C9– without disturbing the helix structure. Moreover, considering that protons A5H2, A6H2, T7H1′, T8H1′, and C9H1′ reside in the minor groove of the helix, we can also conclude that (**7**)Cl_2_ binds within this groove. However, it is crucial to note the significant upfield shifts of DNA protons, T7H6, T8H6, and C9H6, located at the major groove of the helix. Given the DNA structural limitations preventing simultaneous accommodation within both the major and minor grooves, the possibility of non-specific binding in both grooves is a viable scenario. This assumption is supported by the NOE maps of the mixtures, illustrating some cross-peaks between the complex and the DNA protons (Figure 7)

More specifically, in NOESY spectra in r = 1, weak cross-peaks between the A5H2 and A6H2 with the protons of the phenyl ring (a) H_2a6a_ and H_3aH5a_ have been observed, suggesting a close proximity (<4Å) of this segment of (**7**)Cl_2_ to the –A5A6– sequence. This observation is consistent with the upfield shifts of these protons, induced by the positive charge of the {RuCp} unit. The cross-peaks between the protons of the next phenyl rings, (b) and (c), and the T8H1′, C9H1′ or T7H1′ confirms the extension of the ring system of (**7**)Cl_2_ toward the sequence end. Considering that these protons reside in the minor groove of the helix, and their upfield shifts result from the high electron density in the proximity with the aromatic rings (b) and (c), it can be assumed that (**7**)Cl_2_ accommodates in the minor groove. Also, cross-peaks observed between the T7H6 and H_3b5b/3c5c_ and H_2b6b/2c6c_ of the rings (b) and (c) strongly support the statement of a major groove binding. Notably, as shown in Figure 7, in the NOE map of the oligonucleotide (light blue), all anticipated cross-peaks between the nucleotide residues in the sequence duplex (gray) remain intact. This indicates that despite the non-specific binding of (**7**)Cl_2_, the structure of the DNA retains the B-form [41]. Visual representations of the NMR results are presented in Figure 9a.

##### [(*η*^5^-C_5_H_5_)Ru_2_(*η*^6^-*p*-quaterphenyl)]Cl, (**6**)Cl

Similar to (**7**)Cl_2_, the 1HNMR spectrum of the complex (**6**)Cl in an aqueous phosphate buffer closely resembles its [PF_6_] analog. Upon addition of (**6**)Cl to DNA at r = 0.5, an immediate formation of precipitate occurs, which contains almost the entire amount of both DNA and (**6**)Cl. The addition of NaCl until it reaches a concentration of 10 mM results in complete dissolution of the solid, enabling the recording of its NMR spectrum. This phenomenon can be interpreted in terms of electrostatic interactions between the bulky, complex cation and the polyanionic DNA, wherein the addition of NaCl shifts the equilibrium towards the soluble products. However, beyond the r = 0.5, the precipitate does not entirely dissolve, making the examination of the interaction uncertain.

At r = 0.5, broad signals for the protons of the phenyl ring (a, b, c, d) were observed. Thus, the assignments were assisted by the COSY spectrum of the mixture (Figure S13, Table S1). The proton signals from ring (a), *η*^6^-coordinated with the {RuCp} unit, exhibit downfield shifts in the region of 0.02 to 0.14 ppm (Table S1). These shifts are comparable in magnitude to those observed in the case of (**7**)Cl_2_, suggesting an increase in the electron density of the quaterphenyl ring system, probably due to the involvement of the neighboring rings, (b), (c) and (d), in the binding of the complex to DNA. However, all protons of ring (d) show significant upfield shifts, with chemical shift values similar to those observed for ring (a). Thus, the proton chemical shifts for rings (a) and (d) appear at a lower field (Figure S13, Table S1). Such a large shift can be attributed to the close proximity of ring (d) to the aromatic system of DNA bases. Simultaneously, the proton signals of ring (c) shifted more upfield compared to those of ring (b). This progressive upfield shift of the quaterphenyl rings from (b) to (d) indicates a higher binding affinity of the terminal ring (d) towards DNA. However, the bulky shape and the positive charge, derived from the Ru center, suggest that the ring (a) participates in the binding probably through electrostatic interactions.

**Figure 8:**
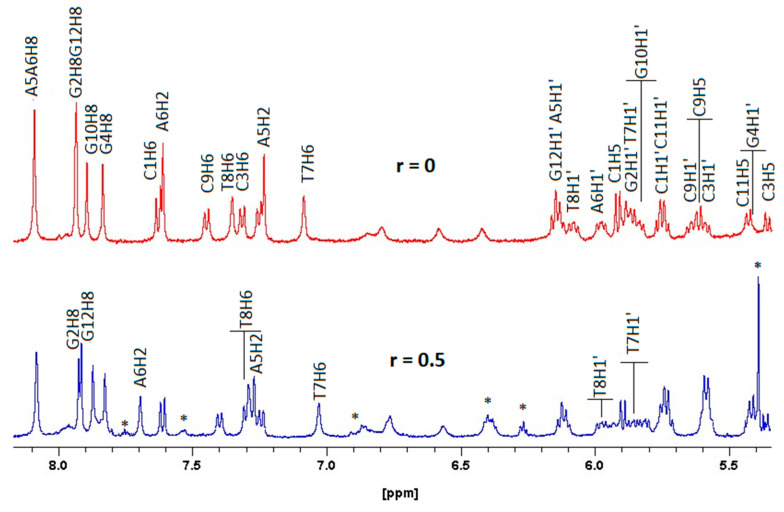
Stacked ^1^H NMR spectra of d(CGCGAATTCGCG)_2_ upon addition of complex (**6**)Cl_2_ at r = 0 and r = 0.5. The complex signals were denoted with an asterisk (*).

**Figure 9:**
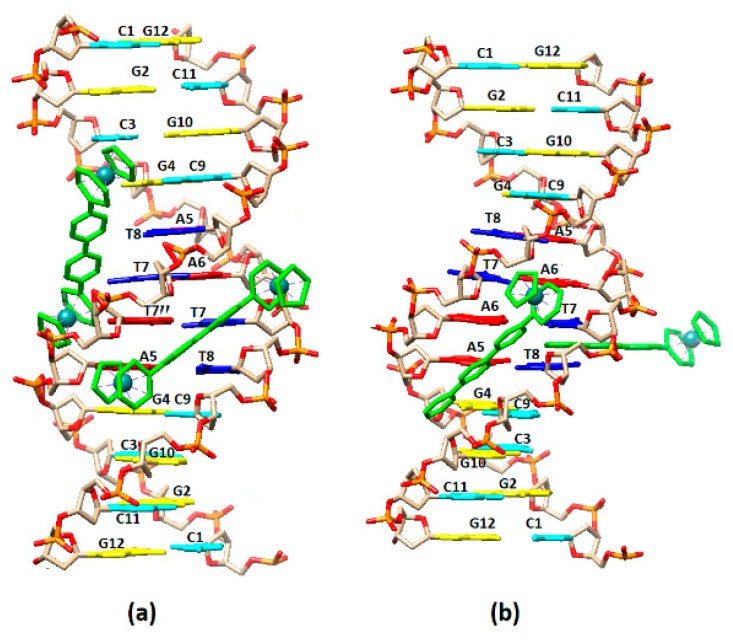
Cartoon representation of the binding of (**a**), (**7**)Cl_2_ and (**b**), (**6**)Cl to the DNA sequence d(5′-CGCGTAGGCC-3′)2 based on the NMR data.

The exchangeable imino and amino protons of the helix, participating in C.-W. hydrogen bonds and keeping the helix strands together, remain almost unchanged, indicating that the helix retains its structure (Table S2). Only the non-hydrogen bonding G4N2**H** or C9N4**H** shifted −0.04 ppm upfield provides evidence for the segment of the sequence where the complex (**6**)Cl binds. Additional insights come from the shifts of the non-exchangeable protons of the DNA. The aromatic protons of the bases T7H6, T8H6, C9H6, and C9H5 shift upfield by −0.05 ppm, indicating a possible binding from the major groove. However, this assumption lacks support from the significant upfield shifts observed for the T7H1′ and T8H1′, located in the helix minor groove, where there is also the A5H2 and A6H2, which shifted downfield by 0.10 and 0.06 ppm, respectively. These contradictory results might be consistent only with non-specific binding of the complex, possibly intercalating between the bases T7 and T8, and binding in the helix minor groove extending from the base A5 towards the sequence’s end (Figure 8, Table S3). Visual representations of the NMR results are presented in Figure 9b.

## 3. Experimental

### 3.1. Materials and Methods

All solvents were of analytical grade and were used without further purification. Dry dichloromethane, acetone, and diethyl ether were prepared as described in the literature [42]. *p*-Terphenyl and biphenyl were purchased from Alfa Aesar (Haverhill, MA, USA), and *p*-quaterphenyl from TCI was used without further purification. The deuterated solvents, acetone-*d*_6_, D_2_O, and dmso-*d*_6_ were purchased from Sigma (Burlington, MA, USA) and were of >99.9% purity. The complex [(C_5_H_5_)Ru(CH_3_CN)_3_](PF_6_) was synthesized according to the literature methods [43]. The deoxynucleotide d(5′-CGCGAATTCGCG-3′) (DNA) was purchased from Eurogentec (Serain, Belgium) and purified by standard purification method. DNA concentrations were quantified by measuring the absorbance at 260 nm. C, H, and N determinations were performed on a PerkinElmer 2400 Series II analyzer (PerkinElmer, Waltham, MA, USA). High-resolution electrospray ionization mass spectra (HR-ESI-MS) were obtained on a Thermo Scientific LTQ Orbitrap XL™ system (Thermo Scientific, Waltham, MA, USA). ^1^H NMR spectra were recorded on a Bruker NEO spectrometer (Bruker, Billerica, MA, USA) operating at a proton frequency of 500.13 MHz and processed using Topspin 4.07 (Bruker Analytik GmbH, Bruker, Billerica, MA, USA). COSY homonuclear experiments were used to assist the assignments of ^1^H signals. The ^1^H NMR spectra of the DNA were recorded in H_2_O:D_2_O, 9:1 (100 mM phosphate buffer, pH = 7.0), at 298 K and referred to the residual HDO peak at *δ* = 4.79 ppm. One-dimensional ^1^HNMR spectra were recorded for samples with a DNA concentration of approximately 0.5 mM, while two-dimensional NMR experiments were performed with more concentrated samples (e.g., 2 mM). Two-dimensional NOESY experiments were performed at a mixing time of 300 ms, and the data sets were acquired with 4096 × 512 complex points at 8 kHz sweep widths in both dimensions. The solvent signal was suppressed with sequence pulse program noesygppr1d. Graphics were produced using UCSF Chimera version 1.16 [44]. The structure of the DNA fragment d(5′-CGCGAATTCGCG-3′)_2_ was downloaded from the PDB database.

### 3.2. Fluorescence Measurements

A fluorescence emission study was carried out using a Jasco FP-8300 fluorimeter (Jasco, Heckmondwike, UK) equipped with a xenon lamp source. All the experiments were performed by using a 10 mm path length cuvette in a 100 mM phosphate buffer at pH 7.0. Successive amounts of each complex from a stock solution of 1 mM were added to 20 μM of d(5′-CGCGAATTCGCG-3′)_2_ saturated with ethidium bromide EtBr (5.07 μM) [45]. The DNA–EtBr sample was titrated with the chloride salts of the complexes (**1**)–(**8**), and the emission spectra were recorded at a wavelength of 500–800 nm with excitation at 480 nm in a 1 cm quartz cell. The excitation and emission slit widths were kept at 5 nm each. All the measurements were recorded after 15 min of incubation at 298 K. Details on the calculations of K_sv_ and K_b_ are presented in the supplementary material.

### 3.3. Cell Culture

The human lung adenocarcinoma cell line A549 and the human fetal lung fibroblasts HFL-1 were cultured in low glucose Dulbecco’s modified Eagle’s medium (DMEM) supplemented with 10% Foetal Bovine Serum, 2 mM L-glutamine, 100 units/mL penicillin and 100 µg/mL streptomycin, in a humidified atmosphere of 5% CO_2_ at 37 °C and were routinely passaged every 2 or 3 days. The medium was changed every other day. When the cells reached confluence, they were detached using 0.2% (*w*/*v*) trypsin and transferred to new culture flasks. The viability was routinely kept at >95%, as assayed by the Crystal Violet exclusion method.

### 3.4. Cell Viability Assay

The A549 tumor cells and HFL-1 fibroblasts were seeded at a density of 6.000 in 96 well plates and, after 24 h, were treated with increasing concentrations of complexes (**6**) and (**7**) for 48 h. Next, cells were fixed in 4% formaldehyde and stained with 0.1% crystal violet dye for 20 min. Cells were washed and left to air dry. Crystal violet was dissolved in 10% acetic acid, and crystal violet absorbance was counted at 595 nm with the use of a Tecan Plate Reader Infinite 200M Pro. The IC_50_ values of the complexes and of cisplatin were determined by fitting Inhibitor concentration versus response curve fit using GraphPad Prism version 9.5.1.

### 3.5. Crystal Structure Analysis

Suitable crystals of compounds [(*η*^5^-C_5_H_5_)Ru(*η*^6^-biphenyl)]PF_6_, (**1**), [(*η*^5^-C_5_H_5_)Ru(*η*^6^-*p*-terphenyl)]PF_6_, (**3**) and [(*η*^5^-C_5_H_5_)_2_Ru_2_(*η*^6^-*p*-terphenyl)](PF_6_)_2_, (**4**) were glued to a thin glass fiber with cyanoacrylate adhesive and placed on the goniometer head. Diffraction data were collected on a Bruker D8 Quest Eco diffractometer, equipped with a Photon II detector and a TRIUMPH (curved graphite) monochromator utilizing Mo Ka radiation (λ = 0.71073 Å) using the APEX 3 software package [46]. The collected frames were integrated with the Bruker SAINT software using a wide-frame algorithm. Data were corrected for absorption effects using the Multi-Scan method (SADABS) [47]. The structures were solved using the Bruker SHELXT Software Package and refined by full-matrix least-squares techniques on F2 (SHELXL 2018/3) [48] via the ShelXle interface [49]. The non-H atoms were treated anisotropically, whereas the organic H atoms were placed in calculated, ideal positions and refined as riding on their respective carbon atoms. In all cases, the cyclopentadienyl moieties were refined as disordered about the Ru–cp_centroid_ axis with twist angles between the two positions spanning the range 21.5–35.2° and occupancies from approximately 70 to 56%. In compounds (**3**) and (**4**), PF_6_**^−^** counter anions were also disordered. PLATON [50] was used for geometric calculations, and X-Seed [51] for molecular graphics. Details on data collection and refinement are presented in Table 4. Full details on the structures can be found in the CIF files in the ESI. CCDC 2306076–2306078 contain the supplementary crystallographic data for this paper. These data can be obtained free of charge via www.ccdc.cam.ac.uk/data_request/cif (accessed on 6 November 2023).

### 3.6. Synthesis of the Complexes

In all the synthetic procedures of the complexes (**1**)–(**8**), dichloromethane and acetone were thoroughly dried and degassed.

*[(η^5^-C_5_H_5_)Ru(η^6^-Biphenyl)]PF_6_*, (**1**): In a 10 mL vial, 10 mg (0.065 mmol) of biphenyl was added to 6 mL CH_2_Cl_2_ and the solution was heated for 5 min at 55 °C. After the complete dissolution of biphenyl, 30 mg (0.07 mmol) of [(*η*^5^-C_5_H_5_)Ru(CH_3_CN)_3_]PF_6_ was added, and the mixture was stirred for 24 h at room temperature. Subsequently, the solvent was removed in vacuo, and the resulting off-white solid was washed with H_2_O (3 × 2 mL) and 100 μL CH_2_Cl_2_. Yield: 60%. Anal. for C_17_H_15_PF_6_Ru: calc.% C, 46.06; H, 4.27; found C, 46.10; H, 4.32. ^1^H NMR: (500 MHz, dmso-*d*_6_, δ in ppm), H_1a_: 7.48 (t, 1H), H_2a6a_: 7.49 (t, 2H), H_3a5a_: 7.73 (d, 2H, ^3^*J*_H-H_ = 7.0 Hz), H_2b6b_: 6.72 (d, 2H, ^3^*J*_H-H_ = 6.0 Hz), H_3b5b_: 6.40 (t, 2H, ^3^*J*_H-H_ = 5.9 Hz), H_1b_: 6.28 (t, 1H, ^3^*J*_H-H_ = 5.6 Hz), CpH: 5.42 (s, 5H). Suitable crystals for X-ray analysis were obtained by dissolution of an amount of (**1**) in 2 mL of CH_2_Cl_2_ and allowed to slowly diffuse with diethyl ether vapors. After a few days, grey crystals appeared, which were collected by filtration, washed with diethyl ether (3 × 2 mL), and dried under vacuum.

*[(η^5^-C_5_H_5_)_2_Ru_2_(η^6^-Biphenyl)](PF_6_)_2_*, (**2**): Complex (**2**) was prepared similarly to (**1**), but approximately 2.5 eq. of [(*η*^5^-C_5_H_5_)Ru(CH_3_CN_3_)]PF_6_ (65 mg, 0.15 mmol) was added. After solvent removal, the resulting white solid was washed with CH_2_Cl_2_ (3 × 2 mL) and H_2_O (3 × 2 mL). Yield: 56%. Anal. for C_22_H_20_P_2_F_12_Ru_2_: calc.% C, 37.33; H, 3.86; found C, 37.30; H, 3.89.^1^H NMR: (500 MHz, dmso-*d*_6_, δ in ppm), H_1a/1b_: 6.36 (t, 2H, ^3^*J*_H-H_ = 5.1 Hz), H_2a6a/2b6b_: 6.42 (t, 4H, ^3^*J*_H-H_ = 6.1 Hz), H_3a5a/3b5b_: 6.73 (d, 4H, ^3^*J*_H-H_ = 6.1 Hz), CpH: 5.55 (s, 10H).

*[(η^5^-C_5_H_5_)Ru(η^6^-p-Terphenyl)]PF_6_*, (**3**): Complex (**3**) was prepared similarly to (**1**). Yield: 45%. Anal. for C_23_H_19_PF_6_Ru: calc.% C, 52.54; H, 4.41; found C, 52.52; H, 2.68. ^1^H NMR: (500 MHz, dmso-*d*_6_, δ in ppm), H_1a_: 6.31 (t, 1H, ^3^*J*_H-H_ = 4.5 Hz), H_2a6a_: 6.44 (t, 2H, ^3^*J*_H-H_ = 6.2 Hz), H_3a5a_, 6.81: (d, 2H, ^3^*J*_H-H_ = 6.2 Hz), H_2b6b_: 7.85 (d, 2H, ^3^*J*_H-H_ = 8.4 Hz), H_3b5b_: 7.81 (d, 2H, ^3^*J*_H-H_ = 8.4 Hz), H_3c5c_: 7.74 (d, 2H, ^3^*J*_H-H_ = 7.9 Hz), H_2c6c_: 7.51 (t, 2H, ^3^*J*_H-H_ = 7.5 Hz), H_1c_: 7.43 (t, 1H, ^3^*J*_H-H_ = 7.5 Hz), CpH: 5.44 (s, 5H). Suitable crystals for X-ray analysis were obtained by dissolution of an amount of (3) in 2 mL of CH_2_Cl_2_ and allowed to slowly diffuse with diethyl ether vapors. After a few days, grey crystals appeared, which were collected by filtration, washed with diethyl ether (3 × 2 mL), and dried under vacuum.

*[(η^5^-C_5_H_5_)_2_Ru_2_(η^6^-p-Terphenyl)](PF_6_)_2_*, (**4**): In a 10 mL vial, 10 mg of *p*-terphenyl (0.04 mmol) was added to 3 mL of CH_2_Cl_2_, and the mixture was heated at 55 °C until it completely dissolved. Then, 1.1 eq. of [(*η*^5^-C_5_H_5_)Ru(CH_3_CN_3_)]PF_6_ (20 mg, 0.045 mmol) dissolved in 1 mL of CH_2_Cl_2_ was added to the reaction mixture, which was stirred for 24 h at room temperature. After the solvent removal, a solution containing 2 eq. (35 mg, 0.08 mmol) of [(*η*^5^-C_5_H_5_)Ru(CH_3_CN_3_)]PF_6_ in 1 mL of acetone was added. The mixture was heated at 55 °C for an additional 24h, filtered, evaporated to dryness, washed with H_2_O (2 mL × 2 times) and CH_2_Cl_2_ (2 mL × 2 times), and dried under vacuum. Yield: 44%. Anal. for C_28_H_24_P_2_F_12_Ru_2_: calc.% C, 42.11; H, 3.98; found C, 42.14; H, 3.95. HR-ESI-MS, positive (*m*/*z*): found. 243.3142, calc. 243.3132 for [C_28_H_24_Ru_2_]^2+^. ^1^H NMR: (500 MHz, dmso-*d*_6_, δ in ppm), H_1a/1c_: 6.33 (t, 2H, ^3^*J*_H-H_ = 5.6 Hz), H_2a6a/2c6c_: 6.45 (t, 4H, ^3^*J*_H-H_ = 5.7 Hz), H_3a5a/3c5c_: 6.78 (d, 4H, *^3^J_H_*_-H_ = 6.0 Hz), H_2b6b/3b5b_: 7.83 (s, 4H), CpH: 5.45 (s, 10H). Suitable crystals for X-ray analysis were obtained by dissolution of an amount of (4) in 2mL of a mixture of methanol:acetone 1:1 and allowed to slow evaporation. After a few days, grey crystals appeared, which were collected by filtration and dried in a vacuum.

*[(η^5^-C_5_H_5_)_3_Ru_3_(η^6^-p-Terphenyl)](PF_6_)_3_*, (**5**): In a 10 mL vial, 10 mg of *p*-terphenyl (0.04 mmol) and 3 mL of CH_2_Cl_2_ were added. The solution was heated at 55 °C until complete dissolution and 3 eq. (52 mg, 0.12 mmol) of [(*η*^5^-C_5_H_5_)Ru(CH_3_CN_3_)]PF_6_ dissolved in 2 mL of CH_2_Cl_2_ was added. The mixture was left to react for 24h at room temperature and then evaporated to dryness, resulting in the formation of a gray solid. Following this, 4 eq. (70 mg, 0.16 mmol) of [(*η*^5^-C_5_H_5_)Ru(CH_3_CN_3_)]PF_6_ dissolved in 3 mL of acetone was added to the crude solid, and the mixture was heated at 55 °C for **a** further 24 h. Then, the solvent was removed, and the solid was washed with H_2_O (2 mL × 2 times) and CH_2_Cl_2_ (2 mL × 2 times). Yield: 38%. Anal. for C_33_H_29_P_3_F_18_Ru_3_: calc.% C, 37.36; H, 3.78; found C, 37.34; H, 3.80. HR-ESI-MS, positive (*m*/*z*): found. 243.3142 calc. 243.3132 for [C_33_H_29_Ru_3_]^3+^. ^1^H NMR: (500 MHz, acetone-*d*_6_, δ in ppm), H_1a/1c_: 6.56 (t, 2H, ^3^*J*_H-H_ = 5.7 Hz), H_2a6a/2c6c_: 6.63 (t, 4H, ^3^*J*_H-H_ = 5.8 Hz), H_3a5a/3c5c_: 6.97 (d, 4H, ^3^*J*_H-H_ = 5.9 Hz), H_2b6b/3b5b_: 7.15 (s, 4H), Cp_1_H: 5.63 (s, 10H), Cp_2_H: 5.73 (s, 5H).

*[(η^5^-C_5_H_5_)Ru(η^6^-p-Quaterphenyl)]PF_6_*, (**6**): Complex (**6**) was prepared similarly to (**1**), but at 20 mg of *p*-quaterphenyl (0.06 mmol) in 40 mL of CH_2_Cl_2_, 0.2 eq. of [(*η*^5^-C_5_H_5_)Ru(CH_3_CN_3_)]PF_6_ (10 mg, 0.02 mmol) in 0.5 mL of CH_2_Cl_2_ was added. Also, after removing the solvent, the crude product was washed with 2 mL of acetone, and the resulting solution was evaporated to dryness. Yield: 36%. Anal. for C_29_H_23_PF_6_Ru: calc.% C, 57.49; H, 4.51; found C, 57.51; H, 4.49. HR-ESI-MS, positive (*m*/*z*): found. 473.0840, calc. 473.0848 for [C_29_H_23_Ru_2_]^+^. ^1^H NMR: (500 MHz, dmso-*d*_6_, δ in ppm), H_1a_: 6.32 (t, 1H, ^3^*J*_H-H_ = 5.4 Hz), H_2a6a_: 6.45 (t, 2H, ^3^*J*_H-H_ = 5.8 Hz), H_3a5a_: 6.81 (d, 2H, ^3^*J*_H-H_ = 6.4 Hz), H_2b6b/3b5b_: 7.83 (4H), H_2c6c/3c5c_: 7.86 (4H), H_3d5d_: 7.74 (d, 2H, ^3^*J*_H-H_ = 7.5 Hz), H_2d6d_: 7.50 (t, 2H, ^3^*J*_H-H_ = 7.5 Hz), H_1d_: 7.40 (t, 1H, ^3^*J*_H-H_ = 7.2 Hz), CpH: 5.46 (s, 5H).

*[(η^5^-C_5_H_5_)_2_Ru_2_(η^6^-p-Quaterphenyl)](PF_6_)_2_*, (**7**): In a 50 mL round-bottom flask, 35 mg of *p*-quaterphenyl (0.11 mmol) and 40 mL of CH_2_Cl_2_ were added. The mixture was heated at 55 °C until complete dissolution. Then, 1 mL of a CH_2_Cl_2_ solution containing 0.3 eq. of [(*η*^5^-C_5_H_5_)Ru(CH_3_CN)_3_]PF_6_ (15 mg, 0.35 mmol) was added, and the mixture was allowed to react for 1 h at room temperature. Next, 1.5 eq. [(*η*^5^-C_5_H_5_)Ru(CH_3_CN_3_)]PF_6_ in 0.5 mL of acetone was added, and the mixture was stirred for 24 h at room temperature. Subsequently, the solvent evaporated to dryness, and the grey solid was washed with H_2_O (2 mL × 2 times) and CH_2_Cl_2_ (2 mL × 2 times). Yield: 30%. Anal. for C_34_H_28_P_2_F_12_Ru_2_: calc.% C, 46.66; H, 4.32; found C, 46.68; H, 4.30. HR-ESI-MS, positive (*m*/*z*): found. 320.0133, calc. 320.0100 for [C_34_H_27_Ru_2_]^2+^. ^1^H NMR: (500 MHz, dmso-*d*_6_, δ in ppm), H_a1/d1_: 6.32 (t, 2H, ^3^*J*_H-H_ = 5.7 Hz), H_a2a6/d2d6_: 6.44 (t, 4H, ^3^*J*_H-H_ = 6.1 Hz), H_a3a5/d3d5_: 6.79 (d, 4H, ^3^*J*_H-H_ = 6.2 Hz), H_2b6b/3b5b_ and H_2c6c/3c5c_: 7.86 (s, 8H), CpH: 5.45 (s, 10H).

*[(η^5^-C_5_H_5_)_3_Ru(η^6^-p-Quaterphenyl)](PF_6_)_3_*, (**8**): In a 50 mL round bottom flask, 20 mg of *p*-quaterphenyl (0.06 mmol) and 40 mL of CH_2_Cl_2_ were added. The mixture was heated at 55 °C until complete dissolution. Next, 1 mL of a CH_2_Cl_2_ solution containing 2 eq. of [(*η*^5^-C_5_H_5_)Ru(CH_3_CN)_3_]PF_6_ (52 mg, 0.12 mmol) was added and the mixture was allowed to react for 1 h at room temperature. Subsequently, 3 eq. [(*η*^5^-C_5_H_5_)Ru(CH_3_CN_3_)]PF_6_ (80 mg, 0.18 mmol) dissolved in 1 mL of acetone was added, and the mixture was stirred for 24 h at room temperature. Then, the solvent was evaporated to dryness, and the resulting grey solid was washed with H_2_O (2 mL × 2 times) and CH_2_Cl_2_ (2 mL × 2 times). Yield: 50%. Anal. for C_39_H_33_P_3_F_18_Ru_3_: calc.% C, 41.08; H, 4.05; found C, 41.06; H, 4.07. HR-ESI-MS, positive (*m*/*z*): found. 268.6579 calc. 268.6570 for [C_39_H_33_Ru_3_]^3+^_._^1^H NMR: (500 MHz, acetone-*d*_6_, δ in ppm), H_1a_: 6.61 (t, 1H), H_2a6a_: 7.03 (t, 2H, ^3^*J*_H-H_ = 6.0 Hz), H_3a5a_: 6.63 (d, 2H), H_2b6b_: 7.17 (d, 2H, ^3^*J*_H-H_ = 6.3 Hz), H_3b5b_: 7.09 (d, 2H, ^3^*J*_H-H_ = 6.4 Hz), H_2c6c/3c5c_: 7.99 (s, 4H), H_2d6d_: 6.89, (d, 2H, ^3^*J*_H-H_ = 6.1 Hz), H_3d5d_: 6.58 (t, 2H), H_1d_: 6.48 (t, 1H, ^3^*J*_H-H_ = 5.9 Hz), HCp_1_: 5.67 (s, 5H), HCp_2_: 5.62 (s, 5H), HCp_3_: 5.54 (s, 5H).

## 4. Conclusions

In conclusion, complexes of biphenyl, terphenyl, and quaterphenyl *η*^6^-coordinated with varying numbers of {RuCp} units were successfully synthesized and characterized. The chloride salts of these complexes exhibited stability in aqueous media, enabling the investigation of their interaction with DNA and the study of their cytotoxic activity against the A549 lung cancer cell line.

Fluorescence quenching studies conducted on the d(5′-CGCGAATTCGCG-3′)_2_-EtBr adduct revealed that the mono-coordinated paraphenylenes with one {RuCp} unit displayed strong interactions with the DNA, while the fully coordinated complexes, (**2**)Cl_2_ and (**5**)Cl_2_, engaged in electrostatic interactions. Additionally, we observed a significant influence of the number of connected phenyl rings in the paraphenylene on the binding strength of their complexes. The binding constants of the complexes (**1**)Cl, (**3**)Cl, (**4**)Cl_2_, (**6**)Cl, (**7**)Cl_2_, and (**8**)Cl_3_ suggest their potential role as groove binders rather than intercalators.

The investigation aimed to determine in which groove the binding occurred was enabled by titrating the DNA duplex, d(3′- CGCGAATTCGCG-5′)_2_ with the complexes (**6**)Cl and (**7**)Cl_2_ monitoring by NMR spectroscopy. The study revealed that both complexes bind non-specifically to both the minor and major grooves of the helix. Specifically, (**6**)Cl exhibited partial binding through intercalation between the T7 and T8 bases of the sequence without disrupting the C. –W. hydrogen bonds of the helix. Also, the bulky shape of the {RuCp} *η*^6^-coordinated phenyl ring seemed to facilitate the binding through electrostatic interactions.

Combining these observations with the cytotoxic activity of (**6**)Cl, it may be concluded that its higher DNA affinity results in increased cytotoxicity (IC_50_ for (**6**)Cl: 17.45 ± 2.1 μΜ). On the other hand, although (**7**)Cl_2_ binds to DNA relatively weakly and shows lower cytotoxicity (IC_50_ for (**7**)Cl_2_: 65.83 ± 1.8 μΜ) than (**6**)Cl, it displays greater selectivity toward the cancer cells. These conclusions hold under the condition that the observed cytotoxic activity of the studied complexes is attributable to their binding with DNA.

## Figures and Tables

**Table 1:** Selected geometrical characteristics ^$^ (Å and °) of the cations in the structurally characterized compounds.

	(1) ^ǂ^	(3)	(4)
Mean Ru–C (C_5_)	2.182, 2.148	2.193	2.179
Mean Ru–C (C_6_)	2.198, 2.202	2.208	2.208
Ru–Centroid C_5_	1.784, 1.766	1.829	1.814
Ru–LS plane C_5_	1.782, 1.765	1.829	1.814
Ru–Centroid C_6_	1.693, 1.696	1.700	1.701
Ru–LS plane C_5_	1.693, 1.696	1.700	1.701
Centroid C_5_–Ru–Centroid C_6_	176.77, 175.65	179.43	178.52
Dihedral angle LS planes C_5_ and C_6_A	3.278, 5.712	1.325	1.085
Dihedral angle LS planes C_6_A and C_6_B	39.02, 32.77	39.24	41.74
Dihedral angle LS planes C_6_B and C_6_C		50.93	
Dihedral angle LS planes C_6_A and C_6_C		89.87	

^$^ Ring designations refer to Figure 2. ^ǂ^ The two values correspond to two different molecules in the asymmetric unit.

**Table 2:** Binding parameters of the (**1**)Cl, (**3**)Cl, (**4**)Cl_2_, (**5**)Cl_3_ and (**6**)Cl, (**7**)Cl_2_, and (**8**)Cl_3_ with the DNA duplex d(5′-CGCGAATTCGCG-3′)_2_ at 298 K.

Complex	K_sv_ (10^3^ M^−1^)	K_b_ (10^3^ M^−1^)	n	Quenching (%)
(**1**)Cl	1.81 ± 0.053	2.330 ± 0.001	1.02	24.98
(**3**)Cl	1.04 ± 0.029	3.532 ± 0.001	1.15	26.97
(**4**)Cl_2_	0.83 ± 0.032	0.815 ± 0.001	1.09	11.31
(**6**)Cl	4.30 ± 0.075	8.111 ± 0.001	1.07	44.38
(**7**)Cl_2_	5.91 ± 0.024	5.933 ± 0.001	1.07	56.17
(**8**)Cl_3_	2.66 ± 0.014	2.803 ± 0.121	1.00	36.72

**Table 3:** IC_50_ values of complexes (**6**)Cl, (**7**)Cl_2_, and cisplatin against the human lung cancer cell line A549 and the human fetal lung fibroblasts HFL-1. In parenthesis, the selectivity index (SI) ^a^. Values are given in μM and represent the average of three independent experiments.

Complexes	A549	HFL-1
Cisplatin	5.49 ± 2.1 (0.8)	4.40 ± 2.1
(**6**)Cl	17.45 ± 2.1 (1.1)	19.35 ± 2.1
(**7**)Cl_2_	65.83 ± 1.8 (4.8)	318.7 ± 1.8

^a^ The selectivity index (SI) has been introduced as the ratio of the IC_50_ of a compound against a cancer cell line and its toxicity IC_50_ against a nonmalignant cell line.

**Table 4:** Crystal data and structure refinement for compounds [(*η*^5^-C_5_H_5_)Ru(*η*^6^-biphenyl)]PF_6_, (**1**), [(*η*^5^-C_5_H_5_)Ru(*η*^6^-*p*-terphenyl)]PF_6_, (**3**) and [(*η*^5^-C_5_H_5_)_2_Ru_2_(*η*^6^-*p*-terphenyl)](PF_6_)_2_, (**4**).

Compound	1	3	4
Empirical formula	C_34_H_30_F_12_P_2_Ru_2_	C_23_H_19_F_6_PRu	C_28_H_24_F_12_P_2_Ru_2_
Formula weight	930.66	541.42	852.55
Temperature (K)	296(2)		
Wavelength (Å)	0.71073		
Crystal system	Triclinic	Monoclinic	Monoclinic
Space group	*P* $\bar{1}$	*C*2/*c*	*P*2_1_/*n*
Unit cell dimensions a, b, c (Å), α, β, γ (°)	10.5346(15), 12.1661(16), 14.845(2), 104.711(8), 104.567(7) 101.525(8)	14.4714(5), 15.8121(6), 18.8661(6), 90, 95.535(1), 90	10.5652(6), 13.4766(8), 10.7065(6), 90, 101.663(3), 90
Volume (Å^3^)	1708.9(4)	4296.9(3)	1492.95(15)
Z	2	8	2
Density (calcd.) (g/cm^3^)	1.809	1.674	1.897
Absorption coefficient (mm^−1^)	1.068	0.863	1.213
F(000)	920	2160	836
Crystal size (mm^3^)	0.30 × 0.10 × 0.06	0.60 × 0.50 × 0.50	0.20 × 0.15 × 0.02
θ range for data collection (°)	2.339 to 24.998	2.788 to 24.998	2.896 to 24.998
Index ranges	−12 ≤ h ≤ 12, −14 ≤ k ≤ 14, −17 ≤ l ≤ 17	−17 ≤ h ≤ 17, −18 ≤ k ≤ 18, −20 ≤ l ≤ 22	−12 ≤ h ≤ 12, −16 ≤ k ≤ 16, −12 ≤ l ≤ 12
Reflections collected	90,759	62,541	43,243
Independent reflections	6029 [R_int_ = 0.1691]	3774 [R_int_ = 0.0334]	2629 [R_int_ = 0.1992]
Completeness to θ (%)	99.9	99.4	99.9
Refinement method	Full-matrix least-squares on F^2^		
Data/restraints/parameters	6029/312/525	3774/72/310	2629/258/291
Goodness-of-fit	1.004	1.091	1.069
Final R indices [I > 2σ(I)]	R_obs_ = 0.0403, wR_obs_ = 0.0910	R_obs_ = 0.0417, wR_obs_ = 0.1010	R_obs_ = 0.0603, wR_obs_ = 0.1053
R indices [all data]	R_all_ = 0.0791, wR_all_ = 0.1031	R_all_ = 0.0440, wR_all_ = 0.1021	R_all_ = 0.0955, wR_all_ = 0.1151
Largest diff. peak and hole (e·Å^−3^)	0.508 and −0.622	1.256 and −0.719	0.529 and −0.781

R = Σ||F_o_| − |F_c_||/Σ|F_o_|, wR = {Σ[w(|F_o_|^2^ − |F_c_|^2^)^2^]/Σ[w(|F_o_|^4^)]}^1/2^ and w = 1/[σ^2^(Fo^2^) + (αP)^2^ + βP] where P = (Fo^2^ + 2Fc^2^)/3 where α and β are: 1, 0.0521 and 0; 3, 0.0341 and 24.7862; 4, 0.0378 and 5.0600, respectively.

## Data Availability

Data are contained within the article and supplementary materials.

## Supplementary Materials

The following supporting information can be downloaded at https://www.mdpi.com/article/10.3390/molecules29010017/s1, Figure S1. HR-ESI-MS spectrum of complex (**4**) (PF_6_)_2_. Figure S2. HR-ESI-MS spectrum of complex (**5**) (PF_6_)_3_, Figure S3. HR-ESI-MS spectrum of complex (**6**) PF_6_. Figure S4. HR-ESI-MS spectrum of complex (**7**) (PF_6_)_2_. Figure S5. HR-ESI-MS spectrum of complex (**8**) (PF_6_)_3_. Figure S6. Stern–Volmer plots for the interaction of complexes with DNA–EtBr at 298 K. (A) (**1**)Cl, (B) (**3**)Cl, (C) (**4**)Cl_2_, (D) (**6**)Cl, (E) (**7**)Cl_2_, (F) and (**8**)Cl_3_. Figure S7. The double-log plots of complexes quenching effect on d(CGCGAATTCGCG)_2_-EtBr system fluorescence at 298 K. (A) (**1**)Cl, (B) (**3**)Cl, (C) (**4**)Cl_2_, (D) (**6**)Cl, (E) (**7**)Cl_2_, (F) (**8**)Cl_3_. Figure S8. ^1^H NMR spectra (dmso-d_6_, 298 K) of the (a) free terphenyl and (b)–(d) the complexes (**3**)–(**5**), with structures numbering and assignments. Figure S9. ^1^H NMR Spectra of d(CGCGAATTCGCG)_2_ in H_2_O/D_2_O 9:1 (buffer phosphate 100 mM, pH = 7.0) at 298 K, 500 MHz. Figure S10. ^1^H NMR Spectra of complex (**6**) in H_2_O/ D_2_O 9:1 (buffer phosphate 100 mM, pH = 7.0) at 298 K, 500 MHz. Figure S11. ^1^H NMR Spectra of complex (**7**) in H_2_O/D_2_O 9:1 (buffer phosphate 100 mM, pH = 7.0) at 298 K, 500 MHz. Figure S12. ^1^H NMR Spectra of d(CGCGAATTCGCG)_2_ upon addition of complex (**6**)Cl at r = 0.5 in H_2_O/D_2_O 9:1 (buffer phosphate 100 mM, pH = 7.0) at 298 K, 500 MHz. Figure S13. COSY spectrum of the DNA and (**6**)Cl mixture at r = 0.5 (298K, pH =7.0, 100 mM phosphate buffer, NaCl 10 mM) with the complex protons assignment through scalar couplings. The numbered structure of the complex is depicted, with rings highlighted in red indicating those that coordinated with a {RuCp} unit. Figure S14. ^1^H–^1^H NOESY NMR Spectra of d(CGCGAATTCGCG)_2_ upon addition of complex (**6**)Cl at r = 0.5 in H=O/D_2_O 9:1 (buffer phosphate 100 mM, pH = 7.0) at 298 K, 500 MHz. Figure S15. ^1^H NMR Spectra of d(CGCGAATTCGCG)_2_ upon addition of complex (**7**)Cl at r = 0.5 in H_2_O/ D_2_O 9:1 (buffer phosphate 100 mM, pH = 7.0) at 298 K, 500 MHz. Figure S16. COSY spectrum of the DNA and (**7**)Cl_2_ mixture at r = 0.5 (298K, pH =7.0, 100 mM phosphate buffer) with the complex protons assignment through scalar couplings. The numbered structure of the complex is depicted, with rings highlighted in red indicating those coordinated with a {RuCp} unit. Figure S17. ^1^H–^1^H NOESY NMR Spectra of d(CGCGAATTCGCG)_2_ upon addition of complex (**7**)Cl at r = 0.5 in H_2_O/D_2_O 9:1 (buffer phosphate 100 mM, pH = 7.0) at 298 K, 500 MHz. Figure S18. ^1^H NMR Spectra of d(CGCGAATTCGCG)_2_ upon addition of complex (**7**)Cl at r = 1 in H_2_O/D_2_O 9:1 (buffer phosphate 100 mM, pH = 7.0) at 298 K, 500 MHz. Figure S19. ^1^H–^1^H NOESY NMR Spectra of d(CGCGAATTCGCG)_2_ upon addition of complex (**7**)Cl_2_ at r = 1 in H_2_O/D_2_O 9:1 (buffer phosphate 100 mM, pH = 7.0) at 298 K, 500 MHz. Figure S20. ^1^H NMR Spectra of d(CGCGAATTCGCG)_2_ upon addition of complex (**7**)Cl_2_ at r = 2 in H_2_O/D_2_O 9:1 (buffer phosphate 100 mM, pH = 7.0) at 298 K, 500 MHz. Figure S21. ^1^H–^1^H COSY NMR Spectra of d(CGCGAATTCGCG)_2_ upon addition of complex (**7**)Cl_2_ at r = 2 in H_2_O/D_2_O 9:1 (buffer phosphate 100 mM, pH = 7.0) at 298 K, 500 MHz. Figure S22. ^1^H–^1^H NOESY NMR Spectra of d(CGCGAATTCGCG)_2_ upon addition of complex (**7**)Cl_2_ at r = 2 in H_2_O/D_2_O 9:1 (buffer phosphate 100 mM, pH = 7.0) at 298 K, 500 MHz. Figure S23. A perspective view of the packing in the unit cell of (**1**). Figure S24. A perspective view of the packing in the unit cell of (**3**). Figure S25. A perspective view of the packing in the unit cell of (**4**). Table S1. ^1^H NMR chemical shifts of the (**6**)Cl (H_2_O:D_2_O, 9:1, 298 K, buffer phosphates 100 mM, pH = 7.0) free (r = 0), and upon the addition to the d(5′-CGCGAATTCGCG-3′)_2_ at r = 0.5. Shifts are denoting in parenthesis (negative sign upfield and positive sign downfield shifts). Table S2. ^1^H NMR chemical shifts of the exchangeable imino and amino protons of the free d(5′-CGCGAATTCGCG-3′)_2_ (H_2_O:D_2_O, 9:1, 298 K, buffer phosphates 100 mM, pH = 7.0), and induced shifts upon the addition (**6**)Cl at r = 0.5. n.o. = not observed. Table S3. Selected ^1^H NMR chemical shifts of the non-exchangeable protons of the free d(5′-CGCGAATTCGCG-3′)_2_ (H_2_O:D_2_O, 9: 1, 298 K, buffer phosphates 100 mM, pH = 7.0), and induced shifts upon the addition (**6**)Cl at r = 0.5. Negative sign for upfield shifts and positive sign for downfield shifts (in parenthesis). In bold indicated shifts which are higher than 0.05 ppm. Table S4. ^1^H NMR chemical shifts of the (**7**)Cl_2_ (H_2_O:D_2_O, 9:1, 298 K, buffer phosphates 100 mM, pH = 7.0) free (r = 0), and upon the addition to the d(5′-CGCGAATTCGCG-3′)_2_ at r = 0.5, 1 and 2. Shifts are denoting in parenthesis (negative sign upfield and positive sign downfield shifts). Table S5. ^1^H NMR chemical shifts of the exchangeable imino and amino protons of the free d(5′-CGCGAATTCGCG-3′)_2_ (H_2_O:D_2_O, 9:1, 298 K, buffer phosphates 100 mM, pH = 7.0), and induced shifts upon the addition (**7**)Cl_2_ at r = 0.5, 1 and 2. n.o. = not observed. Table S6. Selected ^1^H NMR chemical shifts of the non-exchangeable protons of the free d(5′-CGCGAATTCGCG-3′)_2_ (H_2_O:D_2_O, 9:1, 298 K, buffer phosphates 100 mM, pH = 7.0), and induced shifts upon the addition (**7**)Cl_2_ at r = 0.5, 1 and 2. Negative sign for upfield shifts and positive sign for downfield shifts (in parenthesis). In bold indicated shifts which are higher than 0.05 ppm.
