# Supplementary material for: Synthesis and Characterization of Ruthenium-Paraphenylene-Cyclopentadienyl Full-Sandwich Complexes: Cytotoxic Activity against A549 Lung Cancer Cell Line and DNA Binding Properties
Source: Molecules. 2023 Dec 19;29(1):17. doi: 10.3390/molecules29010017 (PMC10780011; doi:10.3390/molecules29010017)

checkCIF/PLATON report

Structure factors have been supplied for datablock(s) AG12_22_0m

THIS REPORT IS FOR GUIDANCE ONLY. IF USED AS PART OF A REVIEW PROCEDURE FOR PUBLICATION, IT SHOULD NOT REPLACE THE EXPERTISE OF AN EXPERIENCED CRYSTALLOGRAPHIC REFEREE.

No syntax errors found. CIF dictionary Interpreting this report

Datablock: AG12_22_0m

Bond precision:	C-C = 0.0093 A		Wavelength=0.71073
Cell:	a=10.5346(15)	b=12.1661(16)	c=14.845(2)
	alpha=104.711(8)	beta=104.567(7)	gamma=101.525(8)
Temperature:	296 K		

	Calculated	Reported
Volume	1709.0(4)	1708.9(4)
Space group	P -1	P -1
Hall group	-P 1	-P 1
Moiety formula	C17 H15 Ru, F6 P	?
Sum formula	C17 H15 F6 P Ru	C34 H30 F12 P2 Ru2
Mr	465.33	930.66
Dx,g cm-3	1.809	1.809
Z	4	2
Mu (mm-1)	1.068	1.068
F000	920.0	920.0
F000’	915.92	
h,k,lmax	12,14,17	12,14,17
Nref	6034	6029
Tmin,Tmax	0.880,0.938	0.556,0.747
Tmin’	0.726	

Correction method= # Reported T Limits: Tmin=0.556 Tmax=0.747
AbsCorr = MULTI-SCAN

Data completeness= 0.999 Theta(max)= 24.998

R(reflections)= 0.0403(4012) wR2(reflections)= 0.1031(6029)

S = 1.004 Npar= 525

The following ALERTS were generated. Each ALERT has the format
test-name_ALERT_alert-type_alert-level.
Click on the hyperlinks for more details of the test.

Alert level C

```
RINTA01_ALERT_3_C  The value of Rint is greater than 0.12
            Rint given   0.169
PLAT213_ALERT_2_C Atom C2B'            has ADP max/min Ratio .....        3.3 oblate
PLAT234_ALERT_4_C Large Hirshfeld Difference C1A      --C2A      .       0.18 Ang.
PLAT234_ALERT_4_C Large Hirshfeld Difference Ru2      --C2B'     .       0.18 Ang.
PLAT241_ALERT_2_C High   'MainMol' Ueq as Compared to Neighbors of       C14B Check
PLAT242_ALERT_2_C Low    'MainMol' Ueq as Compared to Neighbors of        Ru1 Check
PLAT242_ALERT_2_C Low    'MainMol' Ueq as Compared to Neighbors of        Ru2 Check
PLAT260_ALERT_2_C Large Average Ueq of Residue Including        P1      0.126 Check
PLAT260_ALERT_2_C Large Average Ueq of Residue Including        P2      0.108 Check
PLAT342_ALERT_3_C Low Bond Precision on  C-C Bonds ..............    0.00931 Ang.
PLAT906_ALERT_3_C Large K Value in the Analysis of Variance ......      6.914 Check
PLAT910_ALERT_3_C Missing # of FCF Reflection(s) Below Theta(Min).          5 Note
```

Alert level G

```
PLAT003_ALERT_2_G Number of Uiso or Uij Restrained non-H Atoms ...         20 Report
PLAT020_ALERT_3_G The Value of Rint is Greater Than 0.12 .........      0.169 Report
PLAT045_ALERT_1_G Calculated and Reported Z Differ by a Factor ...          2 Check
PLAT178_ALERT_4_G The CIF-Embedded .res File Contains SIMU Records          2 Report
PLAT188_ALERT_3_G A Non-default SIMU Restraint Value has been used     0.0300 Report
PLAT188_ALERT_3_G A Non-default SIMU Restraint Value has been used     0.0300 Report
PLAT244_ALERT_4_G Low    'Solvent' Ueq as Compared to Neighbors of         P1 Check
PLAT244_ALERT_4_G Low    'Solvent' Ueq as Compared to Neighbors of         P2 Check
PLAT301_ALERT_3_G Main Residue  Disorder .............(Resd  1  )        28% Note
PLAT301_ALERT_3_G Main Residue  Disorder .............(Resd  2  )        28% Note
PLAT720_ALERT_4_G Number of Unusual/Non-Standard Labels ..........         20 Note
PLAT860_ALERT_3_G Number of Least-Squares Restraints .............        312 Note
PLAT883_ALERT_1_G No Info/Value for _atom_sites_solution_primary .     Please Do !
PLAT899_ALERT_4_G SHELXL2018 is Deprecated and Succeeded by SHELXL     2019/3 Note
PLAT909_ALERT_3_G Percentage of I>2sig(I) Data at Theta(Max) Still        43% Note
PLAT961_ALERT_5_G Dataset Contains no Negative Intensities .......     Please Check
PLAT967_ALERT_5_G Note: Two-Theta Cutoff Value in Embedded .res ..       50.0 Degree
PLAT978_ALERT_2_G Number C-C Bonds with Positive Residual Density.          0 Info
```

0 **ALERT level A** = Most likely a serious problem - resolve or explain
0 **ALERT level B** = A potentially serious problem, consider carefully
12 **ALERT level C** = Check. Ensure it is not caused by an omission or oversight
18 **ALERT level G** = General information/check it is not something unexpected

2 ALERT type 1 CIF construction/syntax error, inconsistent or missing data
8 ALERT type 2 Indicator that the structure model may be wrong or deficient
11 ALERT type 3 Indicator that the structure quality may be low
7 ALERT type 4 Improvement, methodology, query or suggestion
2 ALERT type 5 Informative message, check

It is advisable to attempt to resolve as many as possible of the alerts in all categories. Often the minor alerts point to easily fixed oversights, errors and omissions in your CIF or refinement strategy, so attention to these fine details can be worthwhile. In order to resolve some of the more serious problems it may be necessary to carry out additional measurements or structure refinements. However, the purpose of your study may justify the reported deviations and the more serious of these should normally be commented upon in the discussion or experimental section of a paper or in the "special_details" fields of the CIF. checkCIF was carefully designed to identify outliers and unusual parameters, but every test has its limitations and alerts that are not important in a particular case may appear. Conversely, the absence of alerts does not guarantee there are no aspects of the results needing attention. It is up to the individual to critically assess their own results and, if necessary, seek expert advice.

Publication of your CIF in IUCr journals

A basic structural check has been run on your CIF. These basic checks will be run on all CIFs submitted for publication in IUCr journals (*Acta Crystallographica*, *Journal of Applied Crystallography*, *Journal of Synchrotron Radiation*); however, if you intend to submit to *Acta Crystallographica Section C* or *E* or *IUCrData*, you should make sure that full publication checks are run on the final version of your CIF prior to submission.

Publication of your CIF in other journals

Please refer to the *Notes for Authors* of the relevant journal for any special instructions relating to CIF submission.

PLATON version of 06/07/2023; check.def file version of 30/06/2023

Datablock AG12_22_0m - ellipsoid plot

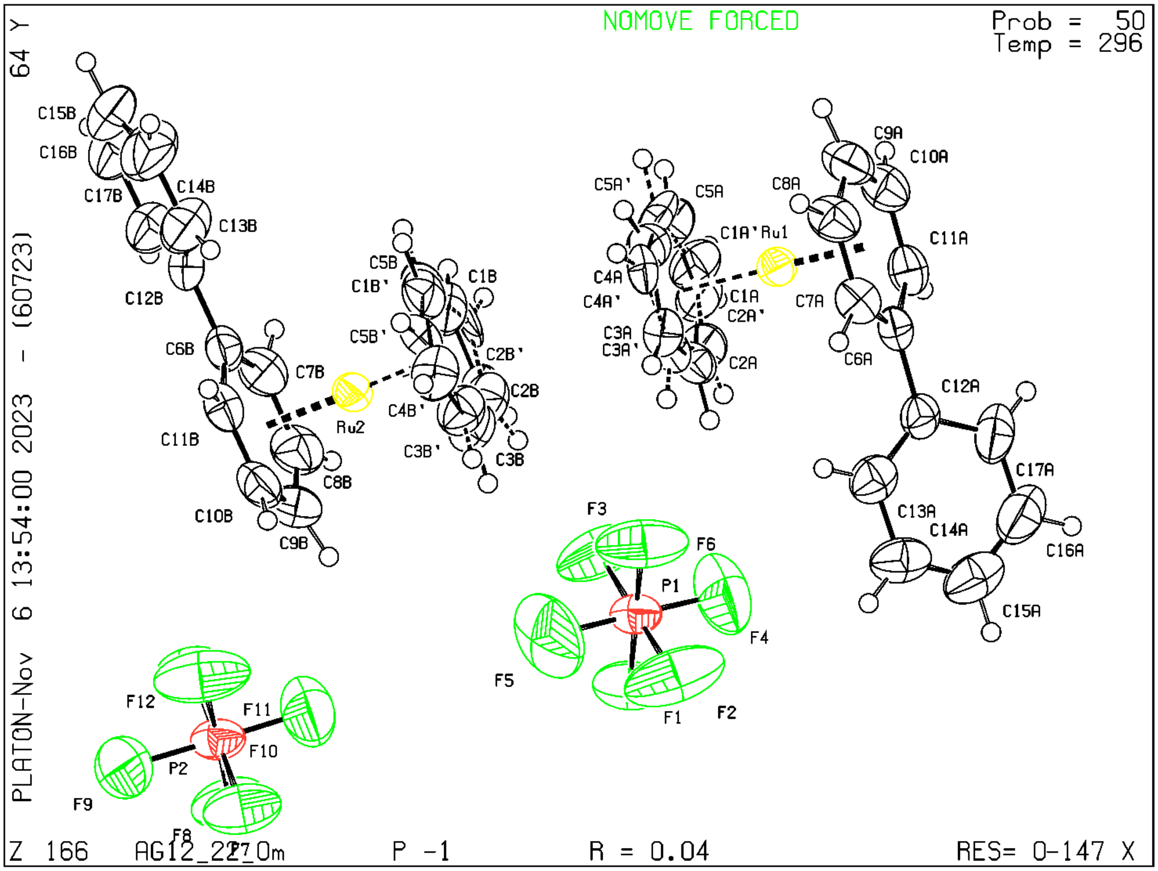

Supplement: Supplementary file 1 [file molecules-29-00017-s001.zip › checkcif_1.pdf]
