# Supplementary material for: Synthesis and Characterization of Ruthenium-Paraphenylene-Cyclopentadienyl Full-Sandwich Complexes: Cytotoxic Activity against A549 Lung Cancer Cell Line and DNA Binding Properties
Source: Molecules. 2023 Dec 19;29(1):17. doi: 10.3390/molecules29010017 (PMC10780011; doi:10.3390/molecules29010017)
Supplement: Supplementary file 1 [file molecules-29-00017-s001.zip › SUPPLEMENTARY VAG..pdf]

Supplementary Material

Evangelia Sifnaiou [a], Theodoros Tsolis [a], Konstantinos Ypsilantis [a], Eugenia Roupakia [c,d], Evangelos Kolettas [c,d], John C. Plakatouras [a,b] and Achilleas Garoufis [a,b]

[a] Laboratory of Inorganic Chemistry, Department of Chemistry, University of Ioannina, Ioannina, Greece

[b] University Research Centre of Ioannina (URCI), Institute of Materials Science and Computing, Ioannina, Greece.

[c] Laboratory of Biology, School of Medicine, Faculty of Health Sciences, University of Ioannina, Greece.

[d] Biomedical Research Division, Institute of Molecular Biology and Biotechnology, Foundation for Research and Technology, 45110 Ioannina, Greece

Table of contents

FIGURE S1. HR-ESI-MS spectrum of complex (**4**) $(PF_6)_2$.

FIGURE S2. HR-ESI-MS spectrum of complex (**5**) $(PF_6)_3$.

FIGURE S3. HR-ESI-MS spectrum of complex (**6**) PF6.

FIGURE S4. HR-ESI-MS spectrum of complex (**7**) $(PF_6)_2$.

FIGURE S5. HR-ESI-MS spectrum of complex (**8**) $(PF_6)_3$.

FIGURE S6. Stern–Volmer plots for the interaction of complexes with DNA–EtBr at 298 K. (A) (**1**)Cl, (B) (**3**)Cl, (C) (**4**)Cl_2, (D) (**6**)Cl, (E) (**7**)Cl_2 , (F) and (**8**)Cl_3.

FIGURE S7. The double-log plots of complexes quenching effect on d(CGCGAATTCGCG)$_2$-EtBr system fluorescence at 298 K. (A) (**1**)Cl, (B) (**3**)Cl, (C) (**4**)Cl_2, (D) (**6**)Cl, (E) (**7**)Cl_2, (F) (**8**)Cl_3.

FIGURE S8. ^{1}H NMR spectra (dmso-d_6, 298 K) of the (a) free terphenyl and (b)-(d) the complexes (**3**)-(**5**), with structures numbering and assignments.

FIGURE S9. ^{1}H NMR Spectra of d(CGCGAATTCGCG)$_2$ in H_2O/ D_2O 9:1 (buffer phosphate 100 mM, pH = 7.0) at 298 K, 500 MHz.

FIGURE S10. ^{1}H NMR Spectra of complex (**6**) in H_2O/ D_2O 9:1 (buffer phosphate 100 mM, pH = 7.0) at 298 K, 500 MHz.

FIGURE S11. ^{1}H NMR Spectra of complex (**7**) in H_2O/ D_2O 9:1 (buffer phosphate 100 mM, pH = 7.0) at 298 K, 500 MHz.

FIGURE S12. ^{1}H NMR Spectra of d(CGCGAATTCGCG)$_2$ upon addition of complex (**6**)Cl at r = 0.5 in H_2O/ D_2O 9:1 (buffer phosphate 100 mM, pH = 7.0) at 298 K, 500 MHz.

FIGURE S13. COSY spectrum of the DNA and (**6**)Cl mixture at r = 0.5 (298K, pH =7.0, 100 mM phosphate buffer, NaCl 10 mM) with the complex protons assignment through scalar couplings. The numbered structure of the complex is depicted, with rings highlighted in red indicating those that coordinated with a {RuCp} unit.

FIGURE S14. ^1H – ^1H NOESY NMR Spectra of d(CGCGAATTCGCG)$_2$ upon addition of complex (**6**)Cl at r = 0.5 in H=O/ D_2O 9:1 (buffer phosphate 100 mM, pH = 7.0) at 298 K, 500 MHz.

FIGURE S15. ^1H NMR Spectra of d(CGCGAATTCGCG)$_2$ upon addition of complex (**7**)Cl at r = 0.5 in H_2O/ D_2O 9:1 (buffer phosphate 100 mM, pH = 7.0) at 298 K, 500 MHz.

FIGURE S16. COSY spectrum of the DNA and (**7**)Cl_2 mixture at r = 0.5 (298K, pH =7.0, 100 mM phosphate buffer) with the complex protons assignment through scalar couplings. The numbered structure of the complex is depicted, with rings highlighted in red indicating those coordinated with a {RuCp} unit.

FIGURE S17. ^1H – ^1H NOESY NMR Spectra of d(CGCGAATTCGCG)$_2$ upon addition of complex (**7**)Cl at r = 0.5 in H_2O/ D_2O 9:1 (buffer phosphate 100 mM, pH = 7.0) at 298 K, 500 MHz.

FIGURE S18. ^1H NMR Spectra of d(CGCGAATTCGCG)$_2$ upon addition of complex (**7**)Cl at r = 1 in H_2O/ D_2O 9:1 (buffer phosphate 100 mM, pH = 7.0) at 298 K, 500 MHz.

FIGURE S19. ^1H – ^1H NOESY NMR Spectra of d(CGCGAATTCGCG)$_2$ upon addition of complex (**7**)Cl_2 at r = 1 in H_2O/ D_2O 9:1 (buffer phosphate 100 mM, pH = 7.0) at 298 K, 500 MHz.

FIGURE S20. ^1H NMR Spectra of d(CGCGAATTCGCG)$_2$ upon addition of complex (**7**)Cl_2 at r = 2 in H_2O/ D_2O 9:1 (buffer phosphate 100 mM, pH = 7.0) at 298 K, 500 MHz.

FIGURE S21. ^1H – ^1H COSY NMR Spectra of d(CGCGAATTCGCG)$_2$ upon addition of complex (**7**)Cl_2 at r = 2 in H_2O/ D_2O 9:1 (buffer phosphate 100 mM, pH = 7.0) at 298 K, 500 MHz.

FIGURE S22. ^1H – ^1H NOESY NMR Spectra of d(CGCGAATTCGCG)$_2$ upon addition of complex (**7**)Cl_2 at r = 2 in H_2O/ D_2O 9:1 (buffer phosphate 100 mM, pH = 7.0) at 298 K, 500 MHz.

FIGURE S23. A perspective view of the packing in the unit cell of (**1**).

FIGURE S24. A perspective view of the packing in the unit cell of (**3**).

FIGURE S25. A perspective view of the packing in the unit cell of (**4**).

TABLE S1. ^1H NMR chemical shifts of the (**6**)Cl (H_2O:D_2O, 9 : 1, 298 K, buffer phosphates 100 mM, pH = 7.0) free (r = 0), and upon the addition to the d(5'-CGCGAATTCGCG-3')$_2$ at r = 0.5. Shifts are denoting in parenthesis (negative sign upfield and positive sign downfield shifts).

TABLE S2. ^1H NMR chemical shifts of the exchangeable imino and amino protons of the free d(5'-CGCGAATTCGCG-3')$_2$ (H_2O:D_2O, 9: 1, 298 K, buffer phosphates 100 mM, pH = 7.0), and induced shifts upon the addition (**6**)Cl at r = 0.5. n.o. = not observed..

TABLE S3. : Selected ^1H NMR chemical shifts of the non-exchangeable protons of the free d(5'-CGCGAATTCGCG-3')$_2$ (H_2O:D_2O, 9: 1, 298 K, buffer phosphates 100 mM, pH = 7.0), and induced shifts upon the addition (**6**)Cl at r = 0.5. Negative sign for upfield shifts and positive sign for downfield shifts (in parenthesis). In bold indicated shifts which are higher than 0.05 ppm.

TABLE S4. ^1H NMR chemical shifts of the (**7**)Cl_2 (H_2O:D_2O, 9 : 1, 298 K, buffer phosphates 100 mM, pH = 7.0) free (r = 0), and upon the addition to the d(5'-CGCGAATTCGCG-3')$_2$ at r = 0.5, 1 and 2. Shifts are denoting in parenthesis (negative sign upfield and positive sign downfield shifts).

TABLE S5. ^{1}H NMR chemical shifts of the exchangeable imino and amino protons of the free d(5'-CGCGAATTCGCG-3')$_2$ (H_2O:D_2O, 9: 1, 298 K, buffer phosphates 100 mM, pH = 7.0), and induced shifts upon the addition (**7**)Cl_2 at r = 0.5, 1 and 2. n.o. = not observed.

TABLE S6. Selected ^{1}H NMR chemical shifts of the non-exchangeable protons of the free d(5'-CGCGAATTCGCG-3')$_2$ (H_2O:D_2O, 9: 1, 298 K, buffer phosphates 100 mM, pH = 7.0), and induced shifts upon the addition (**7**)Cl_2 at r = 0.5, 1 and 2. Negative sign for upfield shifts and positive sign for downfield shifts (in parenthesis). In bold indicated shifts which are higher than 0.05 ppm.

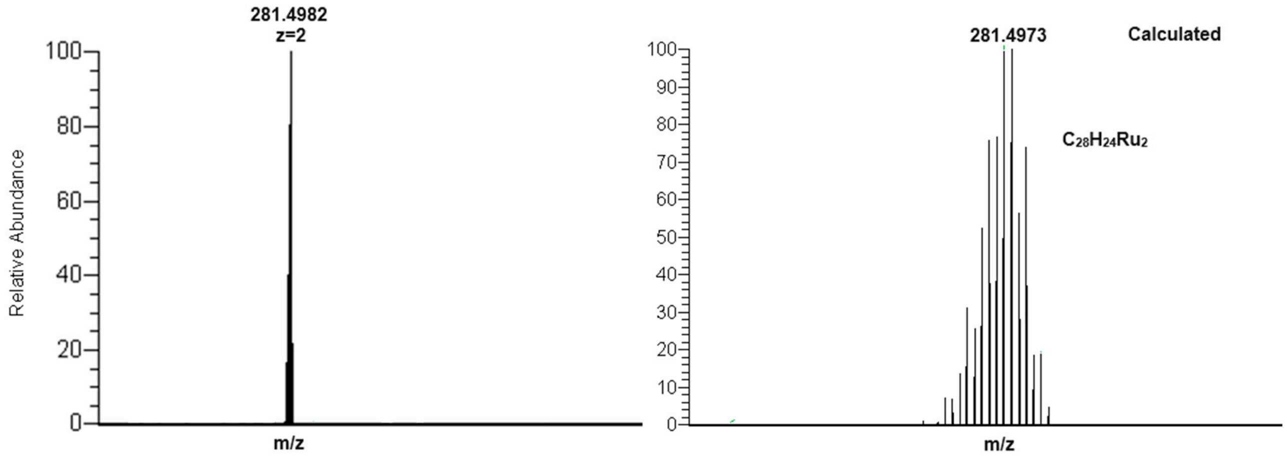

Figure S1. HR-ESI-MS spectrum of complex (**4**) (PF_6)$_2$.

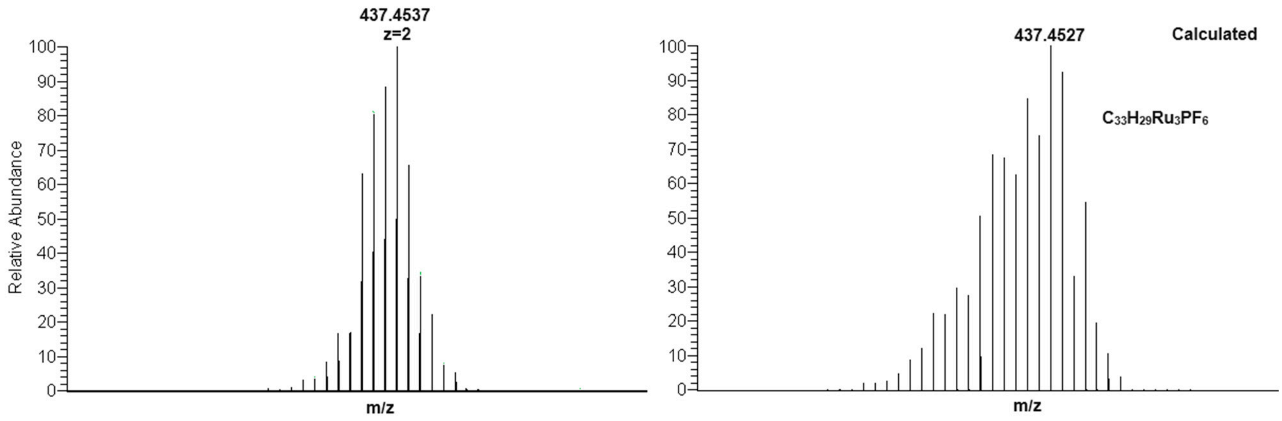

Figure S2. HR-ESI-MS spectrum of complex (**5**) (PF_6)$_3$.

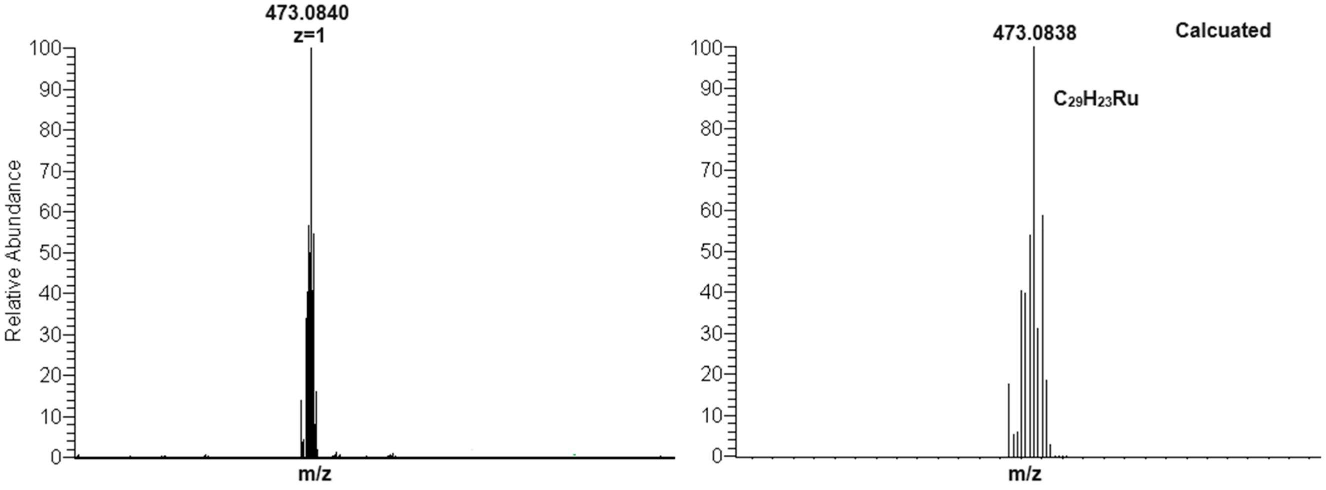

Figure S3. HR-ESI-MS spectrum of complex (**6**) PF_6.

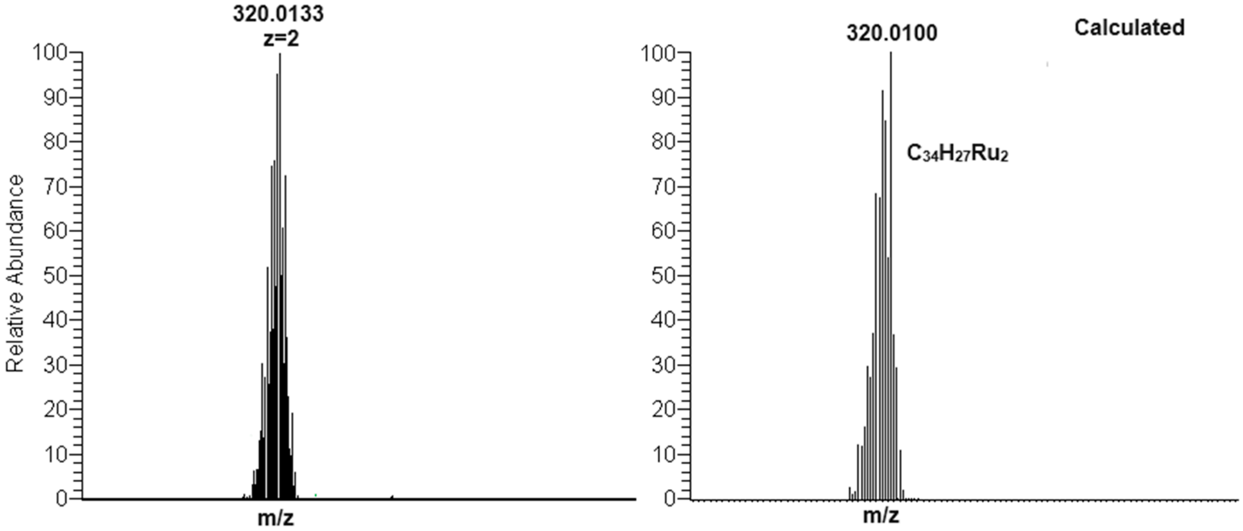

Figure S4. HR-ESI-MS spectrum of complex (**7**) $(PF_6)_2$.

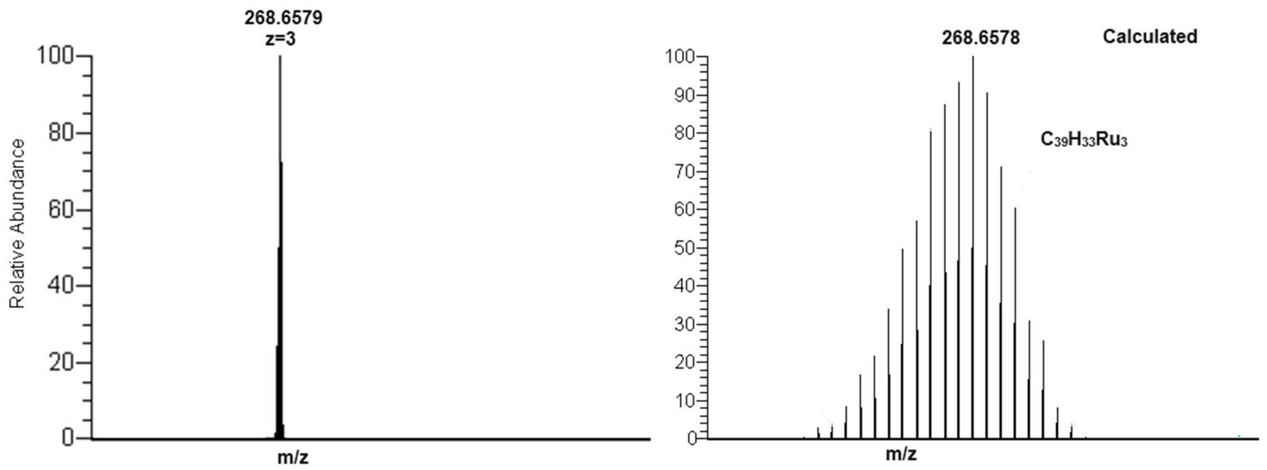

Figure S5. HR-ESI-MS spectrum of complex (**8**) $(PF_6)_3$.

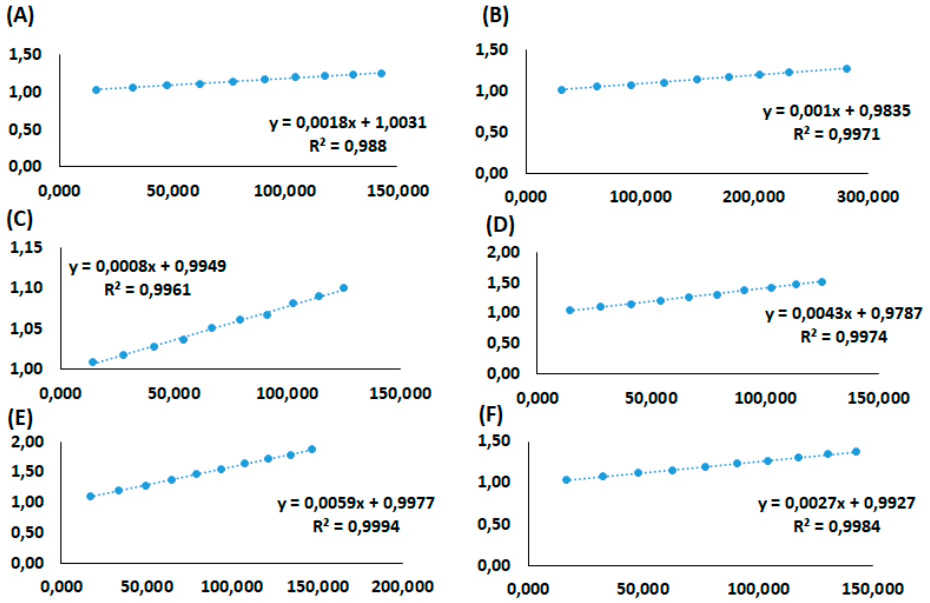

Figure S6. Stern–Volmer plots for the interaction of complexes with DNA–EtBr at 298 K. (A) (**1**)Cl, (B) (**3**)Cl, (C) (**4**)Cl_2, (D) (**6**)Cl, (E) (**7**)Cl_2 , (F) and (**8**)Cl_3.

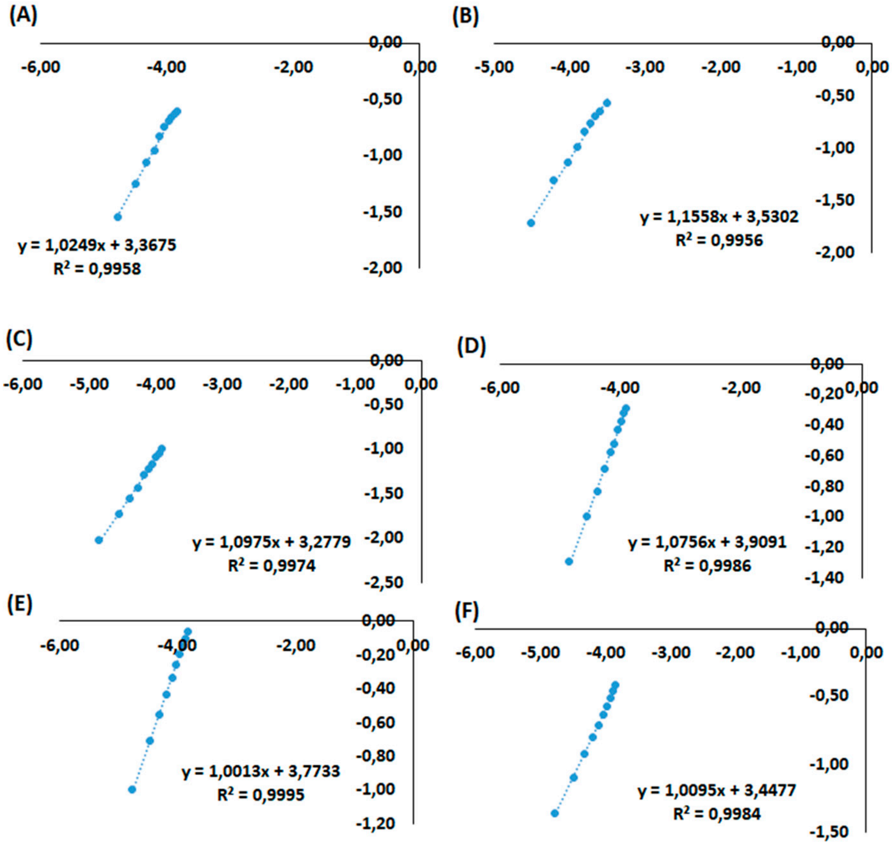

Figure S7. The double-log plots of complexes quenching effect on d(CGCGAATTCGCG)$_2$-EtBr system fluorescence at 298 K. (A) (**1**)Cl, (B) (**3**)Cl, (C) (**4**)Cl_2, (D) (**6**)Cl, (E) (**7**)Cl_2, (F) (**8**)Cl_3.

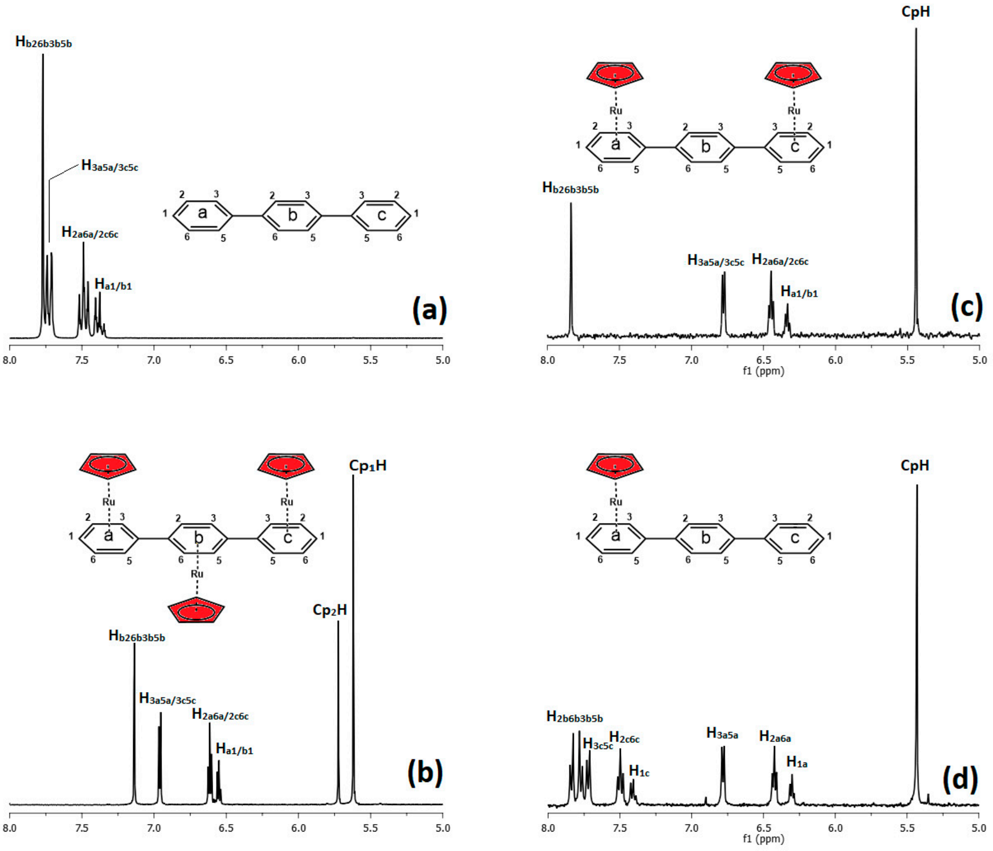

Figure S8. ^{1}H NMR spectra (dmso-d_6, 298 K) of the (a) free terphenyl and (b)-(d) the complexes (**3**)-(**5**), with structures numbering and assignments.

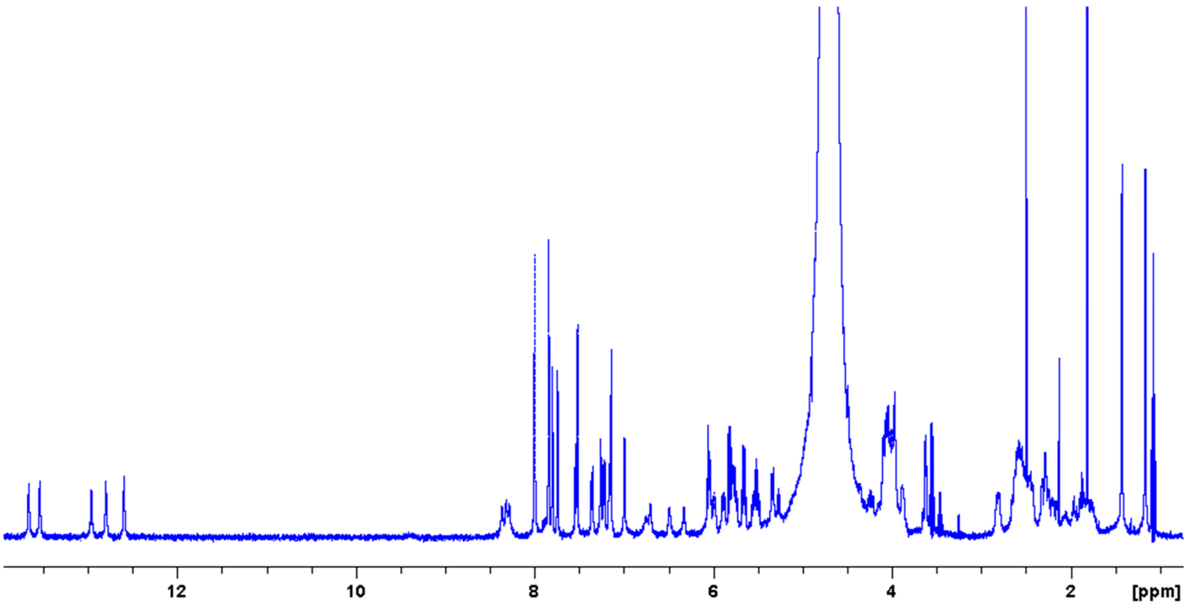

Figure S9. ^{1}H NMR Spectra of d(CGCGAATTCGCG)$_2$ in H_2O/ D_2O 9:1 (buffer phosphate 100 mM, pH = 7.0) at 298 K, 500 MHz.

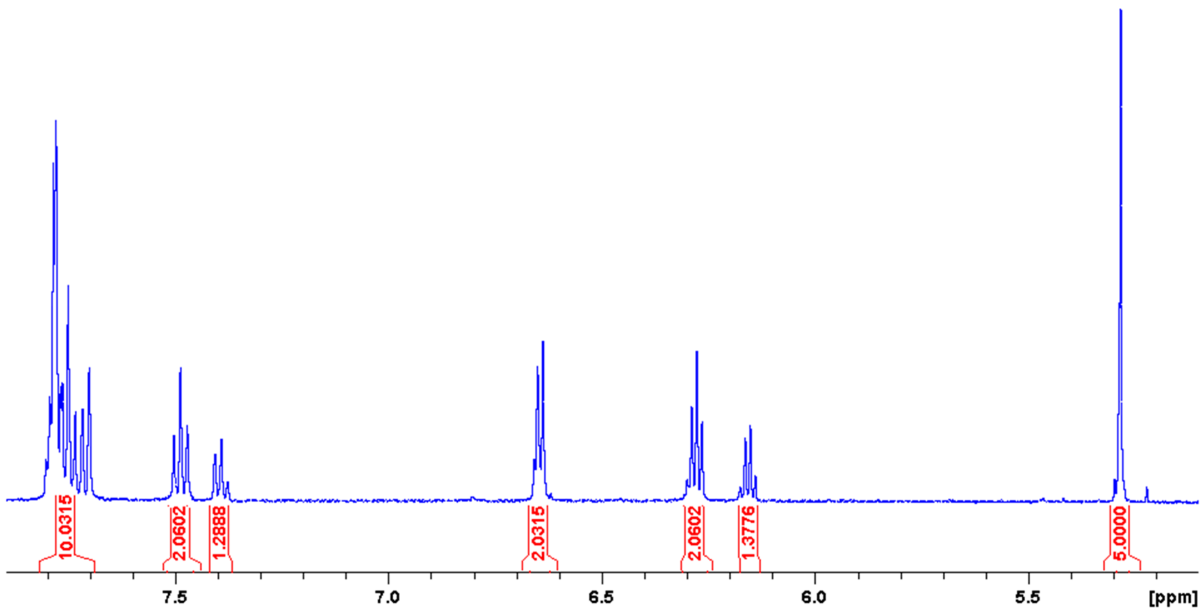

Figure S10. ^{1}H NMR Spectra of complex (**6**) in H_2O/ D_2O 9:1 (buffer phosphate 100 mM, pH = 7.0) at 298 K, 500 MHz.

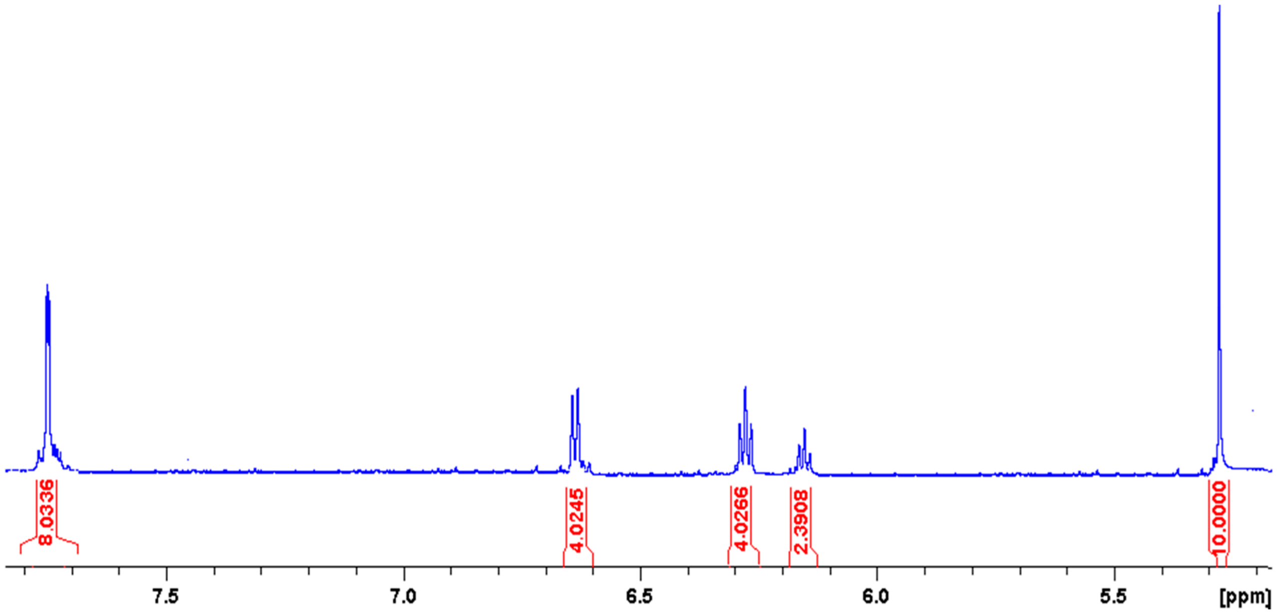

Figure S11. ^{1}H NMR Spectra of complex (**7**) in H_2O/ D_2O 9:1 (buffer phosphate 100 mM, pH = 7.0) at 298 K, 500 MHz.

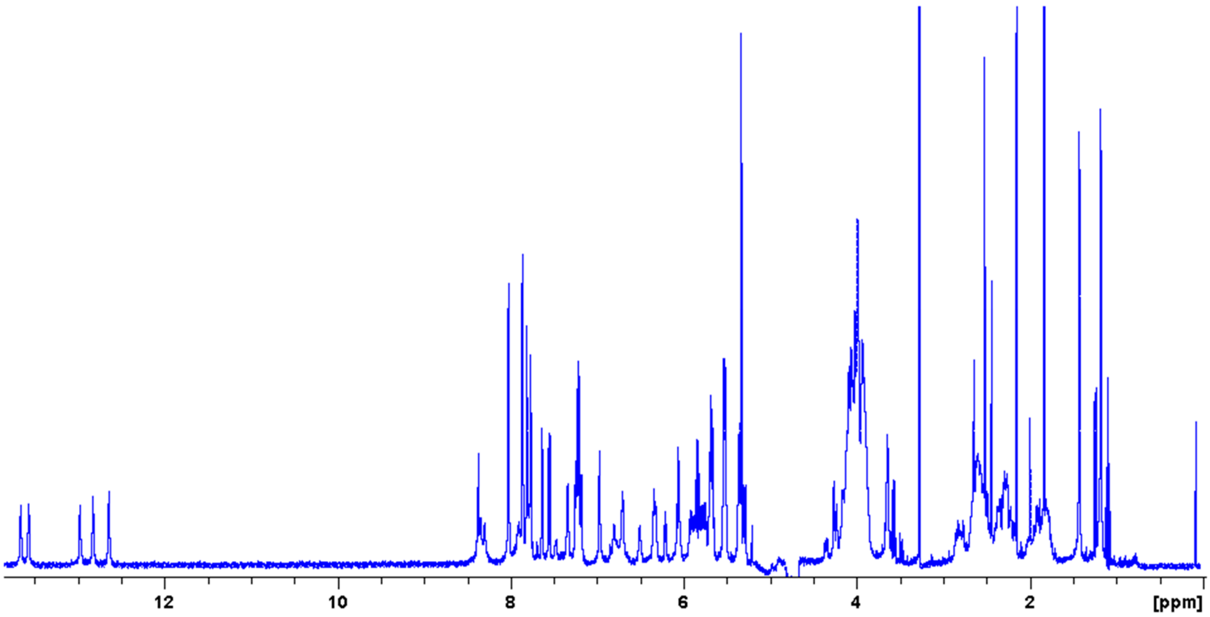

Figure S12. ^{1}H NMR Spectra of d(CGCGAATTCGCG)$_2$ upon addition of complex (**6**)Cl at r = 0.5 in H_2O/ D_2O 9:1 (buffer phosphate 100 mM, pH = 7.0) at 298 K, 500 MHz.

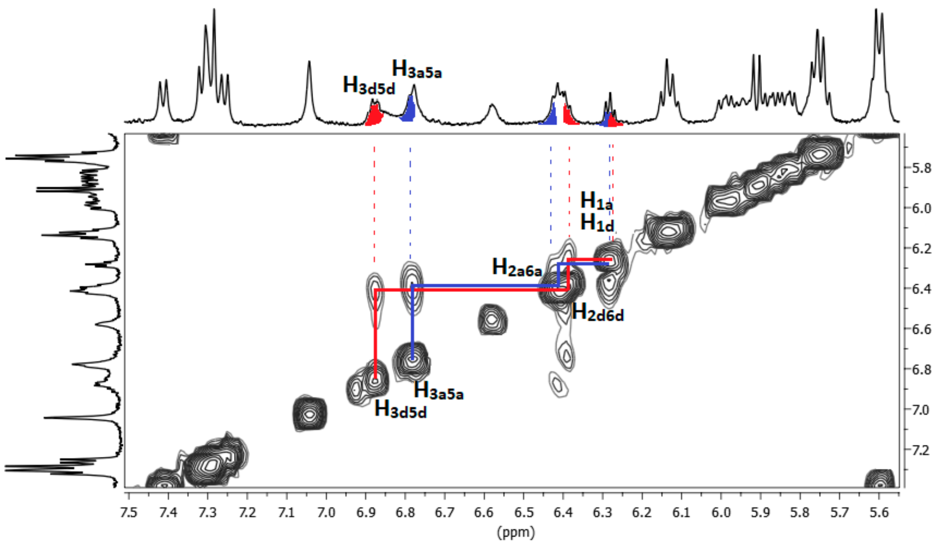

Figure S13. COSY spectrum of the DNA and (**6**)Cl mixture at r = 0.5 (298K, pH =7.0, 100 mM phosphate buffer, NaCl 10 mM) with the complex protons assignment through scalar couplings. The numbered structure of the complex is depicted, with rings highlighted in red indicating those that coordinated with a {RuCp} unit.

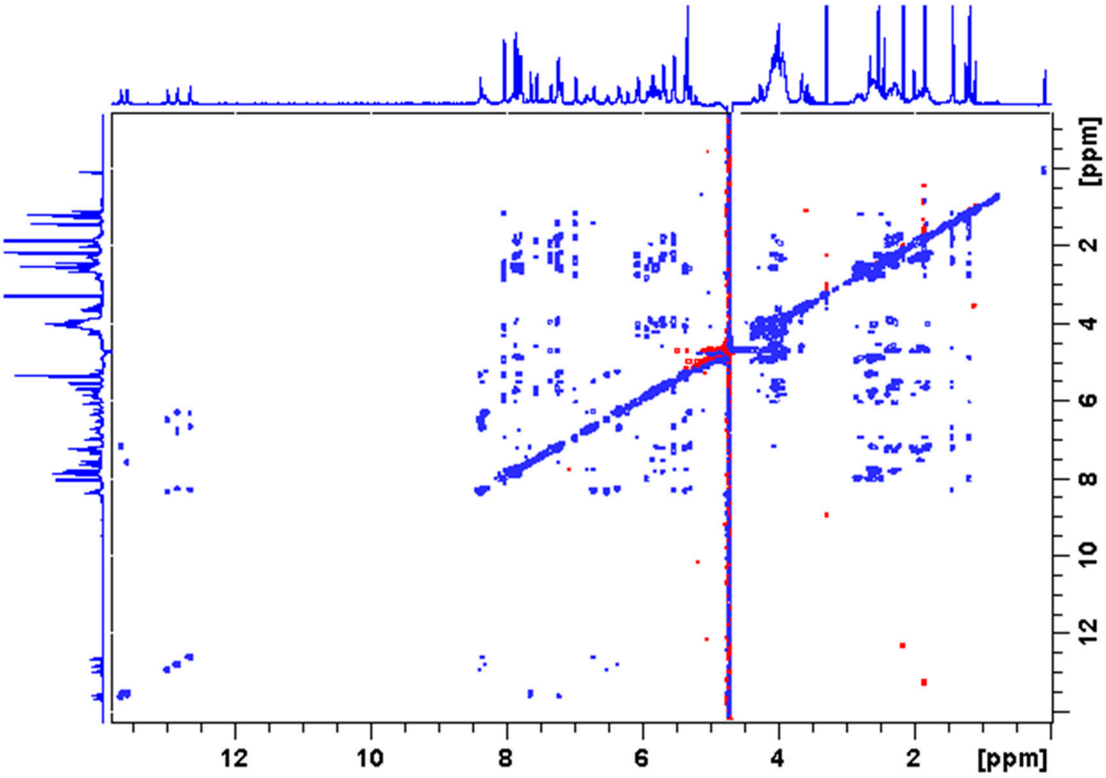

Figure S14. ^{1}H – ^{1}H NOESY NMR Spectra of d(CGCGAATTCGCG)$_2$ upon addition of complex (**6**)Cl at r = 0.5 in H=O/ D_2O 9:1 (buffer phosphate 100 mM, pH = 7.0) at 298 K, 500 MHz.

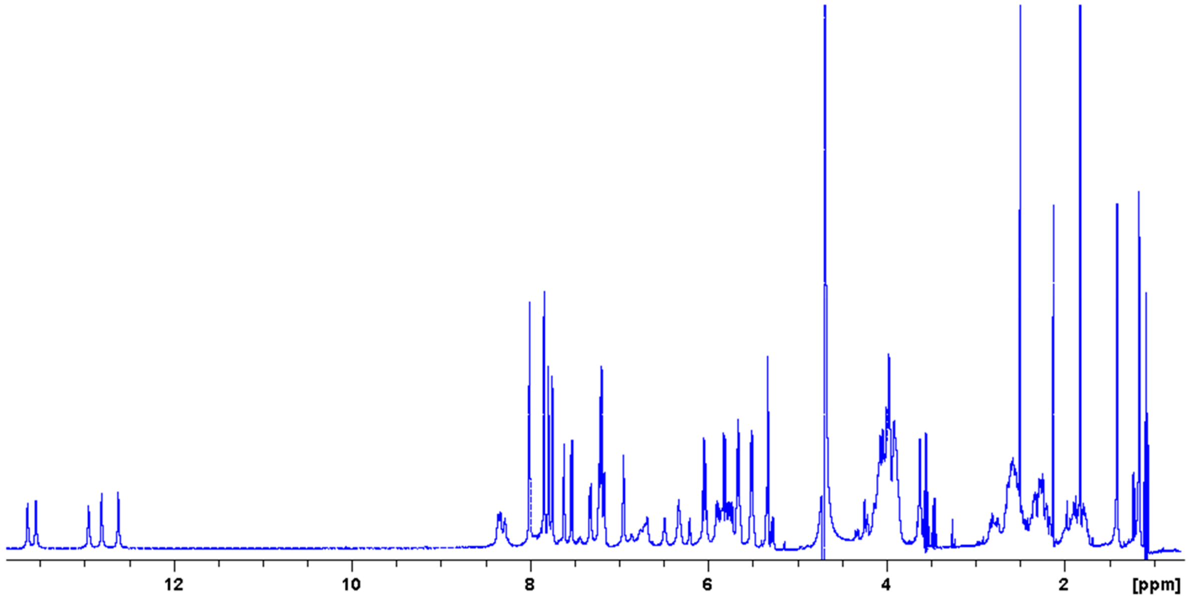

Figure S15. ^{1}H NMR Spectra of d(CGCGAATTCGCG)$_2$ upon addition of complex (**7**)Cl at r = 0.5 in H_2O/ D_2O 9:1 (buffer phosphate 100 mM, pH = 7.0) at 298 K, 500 MHz.

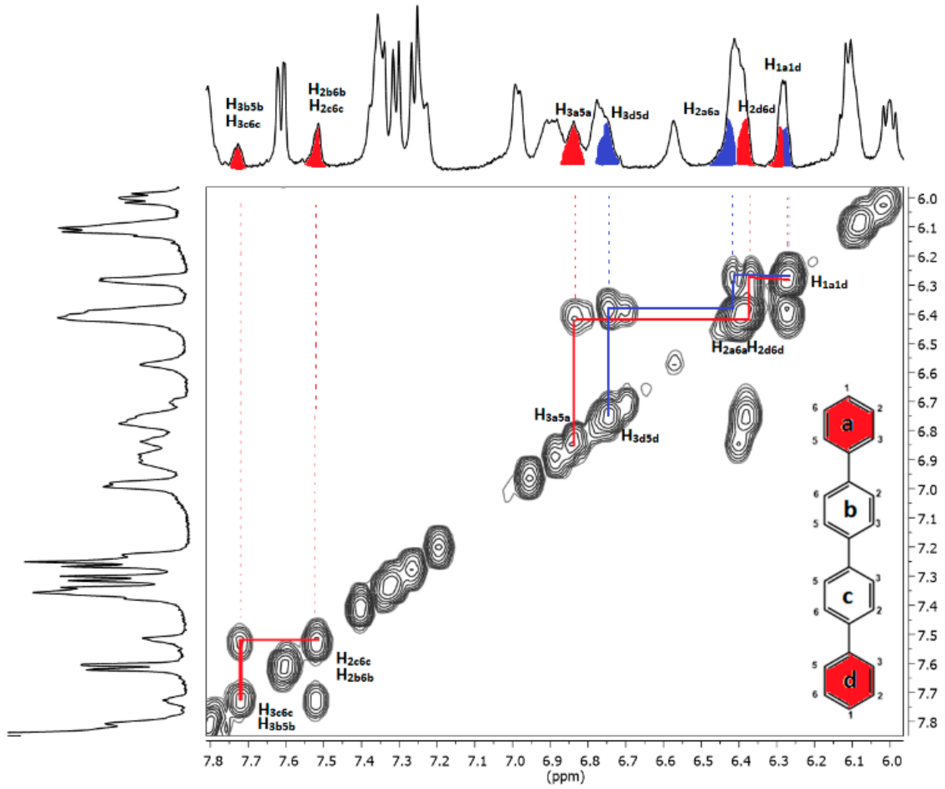

Figure 16. COSY spectrum of the DNA and (**7**)Cl_2 mixture at r = 0.5 (298K, pH =7.0, 100 mM phosphate buffer) with the complex protons assignment through scalar couplings. The numbered structure of the complex is depicted, with rings highlighted in red indicating those coordinated with a {RuCp} unit.

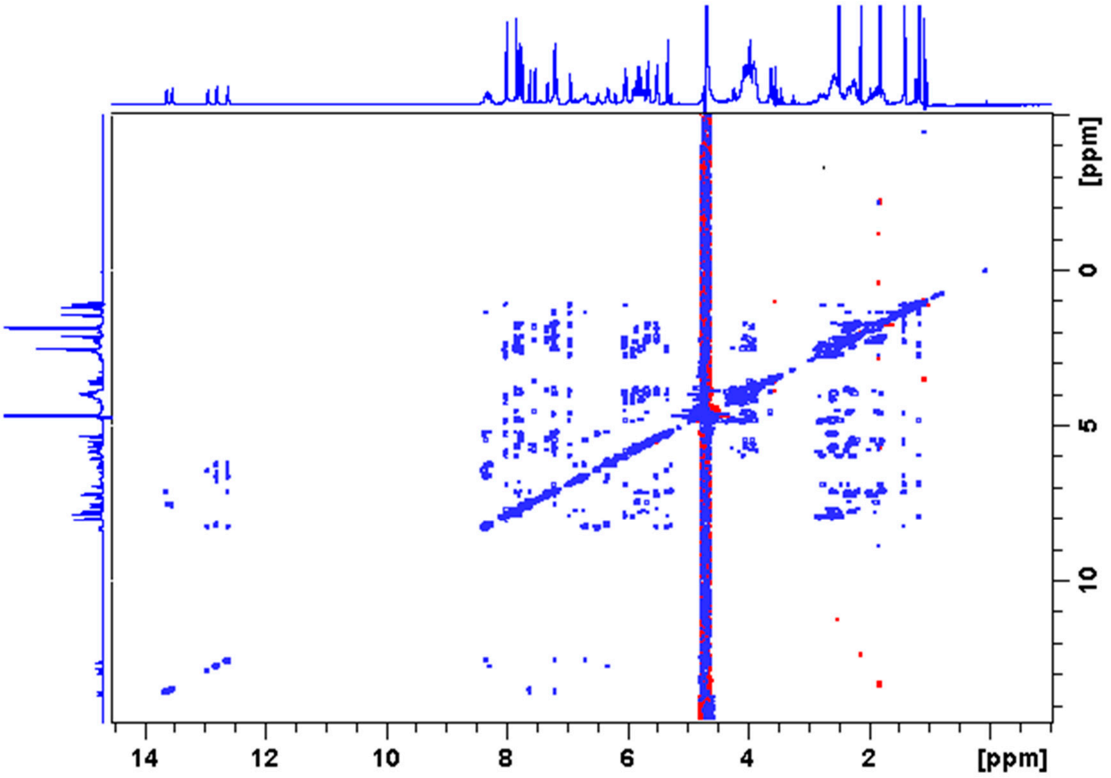

Figure S17. ^{1}H – ^{1}H NOESY NMR Spectra of d(CGCGAATTCGCG)$_2$ upon addition of complex (**7**)Cl at r = 0.5 in H_2O/ D_2O 9:1 (buffer phosphate 100 mM, pH = 7.0) at 298 K, 500 MHz.

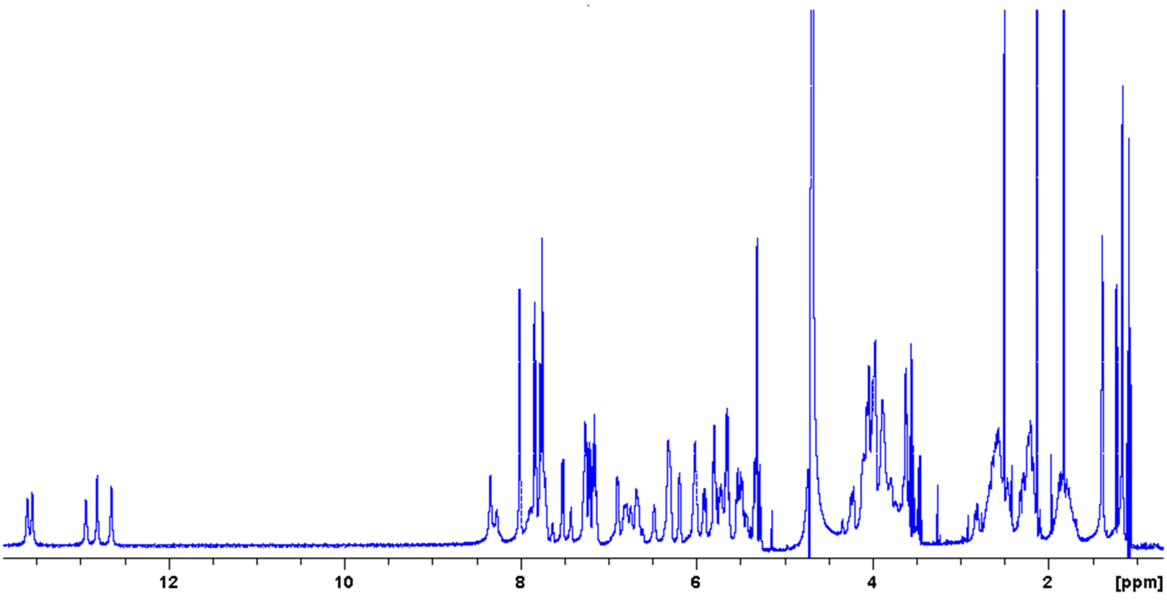

Figure S18. ^{1}H NMR Spectra of d(CGCGAATTCGCG)$_2$ upon addition of complex (**7**)Cl at r = 1 in H_2O/ D_2O 9:1 (buffer phosphate 100 mM, pH = 7.0) at 298 K, 500 MHz.

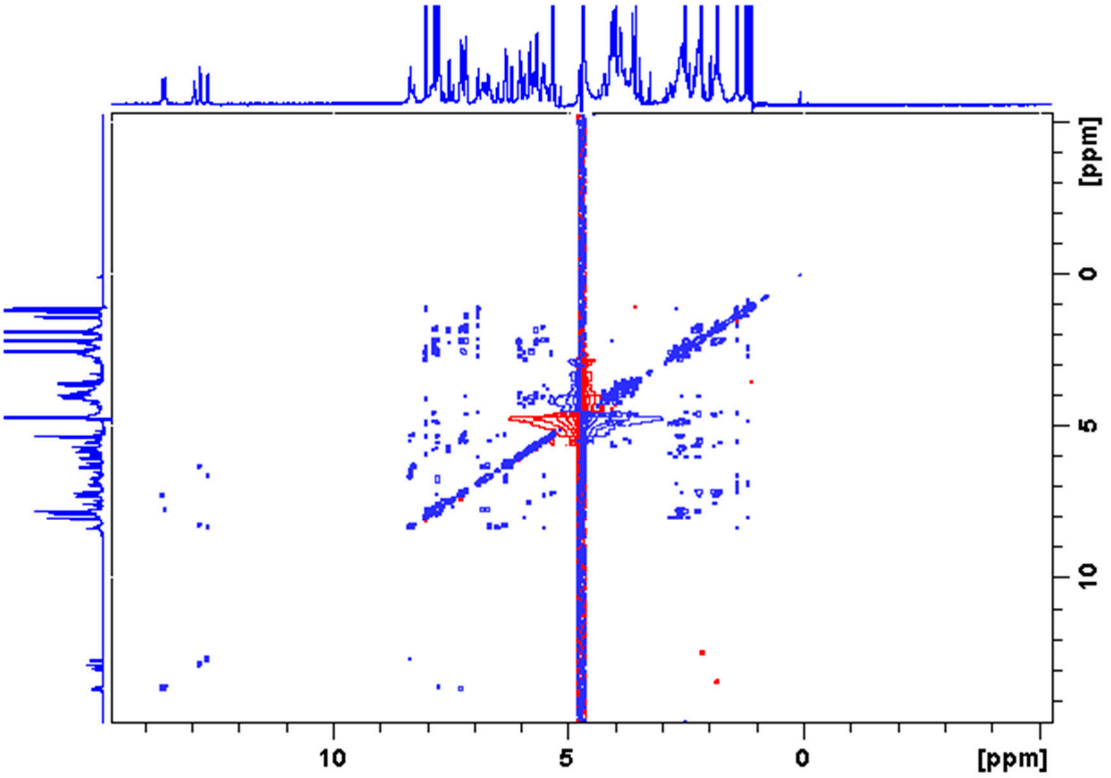

Figure S19. ^1H – ^1H NOESY NMR Spectra of d(CGCGAATTCGCG)$_2$ upon addition of complex (**7**)Cl_2 at r = 1 in H_2O/ D_2O 9:1 (buffer phosphate 100 mM, pH = 7.0) at 298 K, 500 MHz.

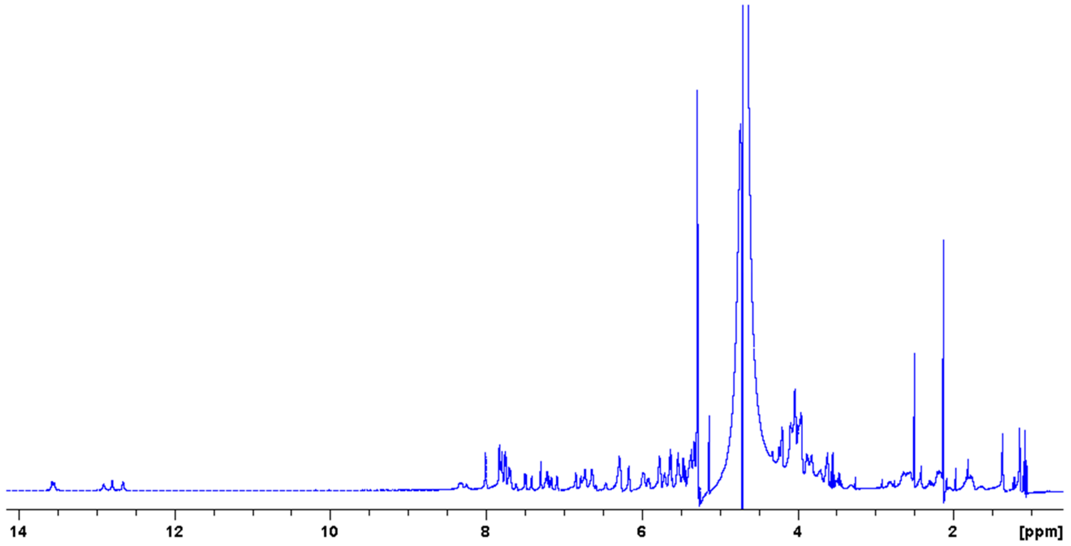

Figure S20. ^1H NMR Spectra of d(CGCGAATTCGCG)$_2$ upon addition of complex (**7**)Cl_2 at r = 2 in H_2O/ D_2O 9:1 (buffer phosphate 100 mM, pH = 7.0) at 298 K, 500 MHz.

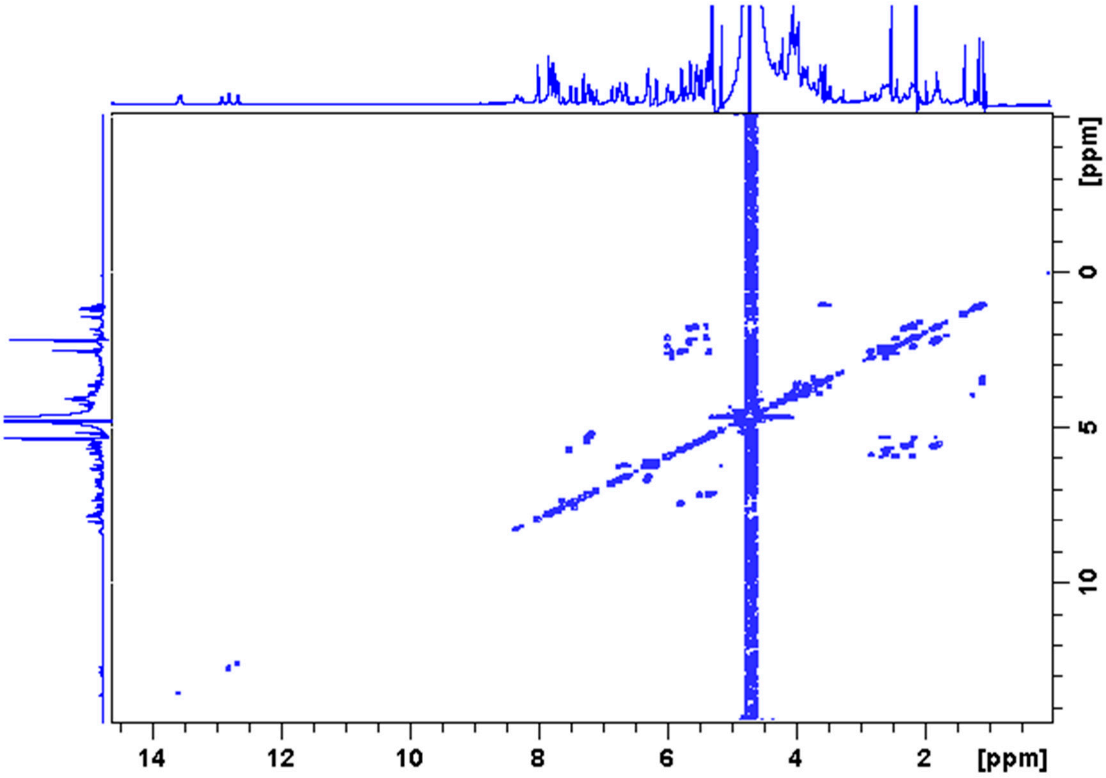

Figure S21. ^{1}H – ^{1}H COSY NMR Spectra of d(CGCGAATTCGCG)$_2$ upon addition of complex (**7**)Cl_2 at r = 2 in H_2O/ D_2O 9:1 (buffer phosphate 100 mM, pH = 7.0) at 298 K, 500 MHz.

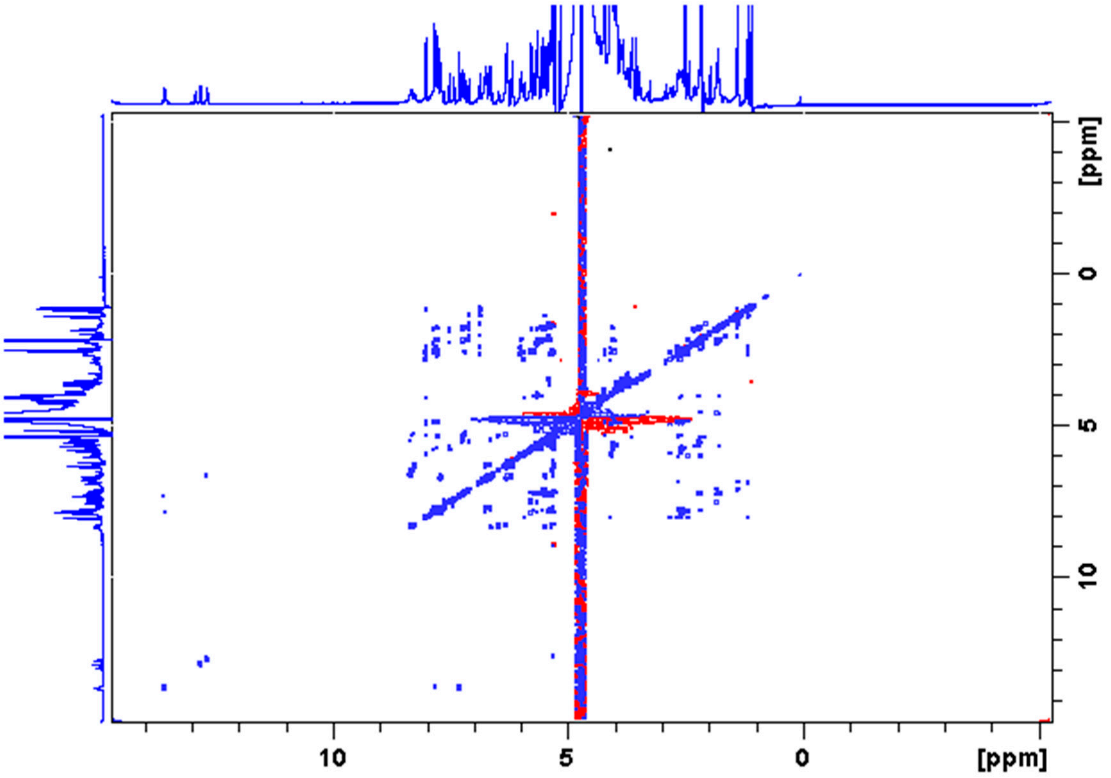

Figure S22. ^1H – ^1H NOESY NMR Spectra of d(CGCGAATTCGCG)$_2$ upon addition of complex (**7**)Cl_2 at r = 2 in H_2O/ D_2O 9:1 (buffer phosphate 100 mM, pH = 7.0) at 298 K, 500 MHz.

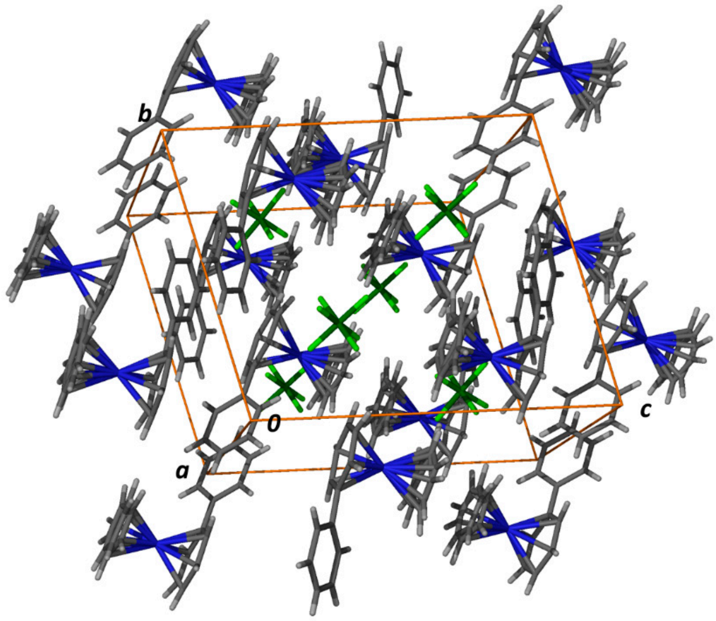

Figure S23. A perspective view of the packing in the unit cell of (**1**).

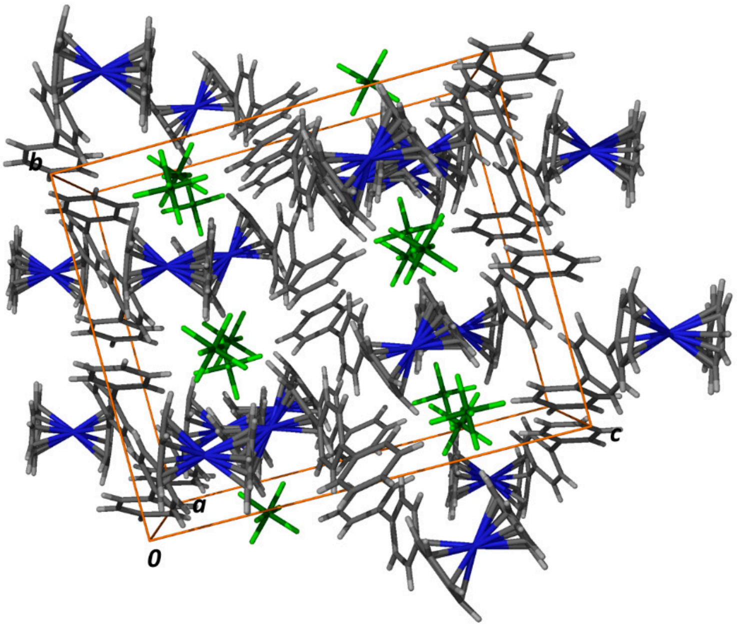

Figure S24. A perspective view of the packing in the unit cell of (**3**).

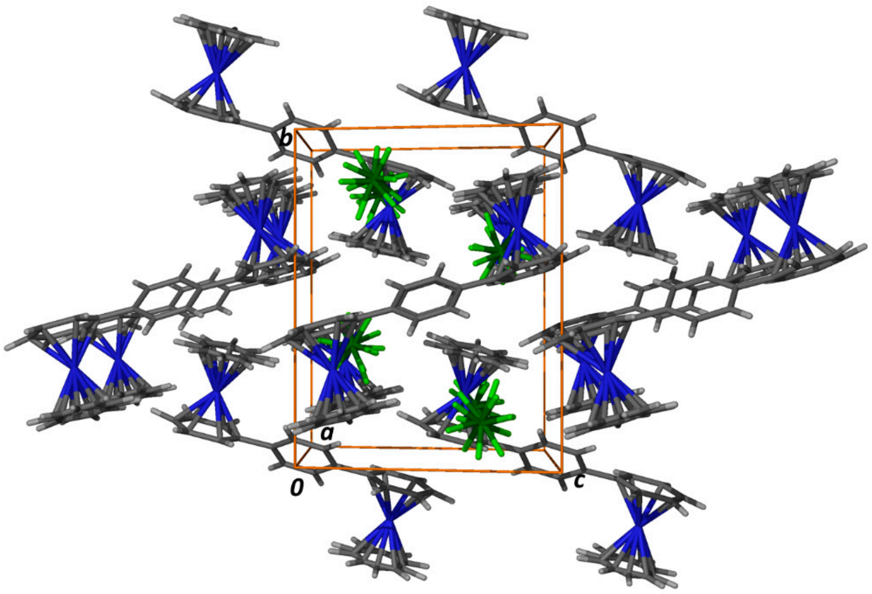

Figure S25. A perspective view of the packing in the unit cell of (**4**).

Table S1. ^{1}H NMR chemical shifts of the (**6**)Cl (H_2O:D_2O, 9 : 1, 298 K, buffer phosphates 100 mM, pH = 7.0) free (r = 0), and upon the addition to the d(5'-CGCGAATTCGCG-3')$_2$ at r = 0.5. Shifts are denoting in parenthesis (negative sign upfield and positive sign downfield shifts).

	H_{1a}	H_{2a6a}	H_{3a5a}	H_{2b6b}	H_{3b5b}	H_{2c6c}	H_{3c5c}	H_{1d}	H_{2d6d}	H_{3d5d}	CpH
r = 0	6.24	6.36	6.73	7.85	7.83	7.85	7.85	7.48	7.57	7.79	5.37
r = 0.5	6.29 (+0.05)	6.38 (+0.02)	6.87 (+0.14)	7.85 (0.00)	7.70 (-0.13	7.75 (-0.10)	7.54 (-0.29)	6.2 (1.19)	6.43 (- 1.14)	6.87 (-0.92)	5.40 (+0.03)

Table S2. ^{1}H NMR chemical shifts of the exchangeable imino and amino protons of the free d(5'-CGCGAATTCGCG-3')$_2$ (H_2O:D_2O, 9: 1, 298 K, buffer phosphates 100 mM, pH = 7.0), and induced shifts upon the addition (**6**)Cl at r = 0.5. n.o. = not observed.

	r = 0			r = 0.5		
	N1/3H	N2/4H	N2/4H*	N1/3H	N2/4H	N2/4H*
T8-A5	13.73			13.73 (0.00)		
T7-A6	13.64			13.64 (0.00)		
G2-C11	13.05	8.44	6.58	13.05 (0.00)	8.44 (0.00)	6.58 (0.00)
G10-C3	12.90	8.39	6.43	12.90 (0.00)	8.39 (0.00)	6.46 (+0.03)
G4-C9	12.71	8.41	6.80	12.71 (0.00)	8.41 (0.00)	6.76 (-0.04)
G12-C1	n.o.	n.o.	n.o.	n.o.	n.o.	n.o.

Table S3. Selected ^{1}H NMR chemical shifts of the non-exchangeable protons of the free d(5'-CGCGAATTCGCG-3')$_2$ (H_2O:D_2O, 9: 1, 298 K, buffer phosphates 100 mM, pH = 7.0), and induced shifts upon the addition (**6**)Cl at r = 0.5. Negative sign for upfield shifts and positive sign for downfield shifts (in parenthesis). In bold indicated shifts which are higher than 0.05 ppm.

	r = 0			r = 0.5		
	H8/6	H5/2 -CH_3	H1'	H8/6	H5/2 -CH_3	H1'
C1	7.63	5.91	5.75	7.63 (0.00)	5.89 (-0.02)	5.74 (-0.01)
G2	7.94		5.88	7.94 (0.00)		5.86 (-0.02)
C3	7.26	5.36	5.56	7.26 (0.00)	5.34 (-0.02)	5.59 (+0.03)
G4	7.84		5.42	7.84 (0.00)		5.41 (-0.01)
A5	8.09	7.24	6.15	8.10 (+0.01)	**7.30 (+0.06)**	6.11 (-0.04)
A6	8.09	7.61	5.98	8.09 (0.00)	**7.71 (+0.10)**	5.98 (0.00)
T7	7.09	1.26	5.88	**7.04 (-0.05)**	1.23 (-0.03)	**5.77 (-0.11)**
T8	7.35	1.52	6.08	**7.30**	1.48	**5.93**

				**(-0.05)**	(-0.04)	**(-0.15)**
C9	7.45	5.62	5.64	7.41 (-0.04)	5.58 (-0.04)	5.60 (-0.04)
G10	7.90		5.83	7.88 (-0.02)		5.81 (-0.02)
C11	7.32	5.43	5.76	7.31 (-0.01)	5.42 (-0.01)	5.77 (+0.01)
G12	7.93		6.13	7.93 (0.00)		6.13 (0.00)

Table S4. ^{1}H NMR chemical shifts of the (**7**)Cl_2 (H_2O:D_2O, 9 : 1, 298 K, buffer phosphates 100 mM, pH = 7.0) free (r = 0), and upon the addition to the d(5'-CGCGAATTCGCG-3')$_2$ at r = 0.5, 1 and 2. Shifts are denoting in parenthesis (negative sign upfield and positive sign downfield shifts).

	(**7**)Cl_2			
	**r = 0**	**r = 0.5**	**r = 1.0**	**r = 2.0**
H_{1a}	6.25	6.31 (+0.06)	6.30 (+0.05)	6.26/6.27 (+0.01)/(+0.02)
$H_{2a/6a}$	6.36	6.43 (+0.07)	6.41 (+0.05)	6.40/6.37 (+0.04)/(+0.01)
$H_{3a/5a}$	6.73	6.96 (+0.23)	6.92 (+0.19)	6.83/6.73 (+0.10)/(0.00)
$H_{2b6b/3b5b}$	7.85	7.54/7.77 (-0.31)/(-0.08)	7.52/7.74 (-0.33)/(-0.11)	7.51/7.71 (-0.34)/(-0.14)
$H_{2c6c/3c5c}$	7.85	7.53/7.75 (-0.32)/(-0.10)	7.53/7.72 (-0.32)/(-0.13)	7.54/7.70 (-0.31)/(-0.14)
H_{1d}	6.25	6.31 (+0.06)	6.30 (+0.05)	6.25 (0.00)
$H_{2d/6d}$	6.36	6.42 (+ 0.06)	6.39 (+0.03)	6.38 (+0.02)
$H_{3d/5d}$	6.73	6.93 (+0.20)	6.78 (+0.05)	6.69 (-0.04)
CpH	5.43	5.42 (-0.01)	5.43 (0.00)	5.41/5.40 (-0.02)/(-0.03)

Table S5. ^{1}H NMR chemical shifts of the exchangeable imino and amino protons of the free d(5'-CGCGAATTCGCG-3')$_2$ (H_2O:D_2O, 9: 1, 298 K, buffer phosphates 100 mM, pH = 7.0), and induced shifts upon the addition (**7**)Cl_2 at r = 0.5, 1 and 2. n.o. = not observed.

	r = 0			r = 0.5			r = 1			r = 2		
	N1/3H	N2/4H	N2/4H*	N1/3H	N2/4H	N2/4H*	N1/3H	N2/4H	N2/4H*	N1/3H	N2/4H	N2/4H*
T8-A5	13.76			13.73			13.69			13.66		
T7-A6	13.63			13.64			13.64			13.64		
G2-C11	13.05	8.44	6.57	13.05	8.44	6.60	13.03	8.44	6.58	13.00	8.43	6.54
G10-C3	12.89	8.39	6.43	12.90	8.38	6.43	12.90	8.37	6.42	12.89	8.35	6.42
G4-C9	12.69	8.41	6.80	12.72	8.43	6.79	12.74	8.45	6.76	12.76	8.45	6.74
G12-C1	n.o.	n.o.	n.o.	n.o.	n.o.	n.o.	n.o.	n.o.	n.o.	n.o.	n.o.	n.o.

Table S6. Selected ^{1}H NMR chemical shifts of the non-exchangeable protons of the free d(5'-CGCGAATTCGCG-3')$_2$ (H_2O:D_2O, 9: 1, 298 K, buffer phosphates 100 mM, pH = 7.0), and induced shifts upon the addition (**7**)Cl_2 at r = 0.5, 1 and 2. Negative sign for upfield shifts and positive sign for downfield shifts (in parenthesis). In bold indicated shifts which are higher than 0.05 ppm.

	r = 0			r = 0.5			r = 1			r = 2		
	H8/6	H5/2 -CH_3	H1'	H8/6	H5/2 -CH_3	H1'	H8/6	H5/2 -CH_3	H1'	H8/6	H5/2 -CH_3	H1'
C1	7.63	5.91	5.75	7.63 (0.00)	5.91 (0.00)	5.75 (0.00)	7.61 (-0.02)	5.90 (-0.01)	5.76 (+0.01)	7.59 (-0.04)	5.88 (-0.03)	5.75 (0.00)
G2	7.94		5.88	7.94 (0.00)		5.87 (-0.01)	7.93 (-0.01)		5.89 (+0.01)	7.90 (-0.04)		5.75 (+0.04)

C3	7.26	5.36	5.56	7.27<br>(+0.01)	5.37<br>(+0.01)	5.56<br>(0.00)	7.26<br>(0.00)	5.37<br>(+0.01)	5.58<br>(+0.02)	7.26<br>(0.00)	5.36<br>(0.00)	5.60<br>(+0.04)
G4	7.84		5.42	7.85<br>(+0.01)		5.42<br>(0.00)	7.84<br>(0.00)		5.42<br>(0.00)	7.84<br>(0.00)		5.44<br>(+0.02)
A5	8.09	7.24	6.15	8.10<br>(+0.01)	**7.29**<br>**(+0.05)**	6.14<br>(-0.01)	8.10<br>(+0.01)	**7.35**<br>**(+0.11)**	6.12<br>(-0.03)	8.10<br>(+0.01)	**7.39**<br>**(+0.15)**	**6.07**<br>**(-0.08)**
A6	8.09	7.61	5.98	8.10<br>(+0.01)	**7.71**<br>**(+0.10)**	5.99<br>(+0.01)	8.09<br>(+0.01)	**7.83**<br>**(+0.22)**	6.00<br>(+0.02)	8.09<br>(0.00)	**7.91**<br>**(+0.30)**	6.00<br>(+0.02)
T7	7.09	1.26	5.88	7.05<br>(-0.04)	1.26<br>(0.00)	**5.77**<br>**(-0.11)**	**6.98**<br>**(-0.11)**	1.25<br>(-0.01)	**5.60**<br>**(-0.28)**	**6.96**<br>**(-0.13)**	1.24<br>(-0.02)	**5.49**<br>**(-0.39)**
T8	7.35	1.52	6.08	7.31<br>(-0.04)	1.50<br>(-0.02)	**5.94**<br>**(-0.14)**	**7.23**<br>**(-0.12)**	1.49<br>(-0.03)	**5.77**<br>**(-0.31)**	**7.18**<br>**(-0.17)**	**1.46**<br>**(-0.06)**	**5.61**<br>**(-0.49)**
C9	7.45	5.62	5.64	7.42<br>(-0.03)	5.60<br>(-0.02)	**5.58**<br>**(-0.06)**	**7.35**<br>**(-0.10)**	5.58<br>(-0.04)	**5.57**<br>**(-0.07)**	**7.33**<br>**(-0.12)**	**5.57**<br>**(-0.05)**	**5.56**<br>**(-0.08)**
G10	7.90		5.83	7.89<br>(-0.01)		5.82<br>(-0.01)	7.87<br>(-0.03)		5.82<br>(-0.01)	**7.85**<br>**(-0.05)**		5.80<br>(-0.03)
C11	7.32	5.43	5.76	7.32<br>(0.00)	5.44<br>(+0.01)	5.76<br>(0.00)	7.31<br>(-0.01)	5.43<br>(0.00)	5.75<br>(-0.01)	7.30<br>(-0.02)	5.43<br>(0.00)	5.74<br>(-0.02)
G12	7.93		6.13	7.93<br>(0.00)		6.14<br>(+0.01)	7.91<br>(-0.02)		6.12<br>(+0.01)	7.89<br>(-0.01)		6.07<br>(-0.06)
