# Supplementary material for: Synthesis and Characterization of Ruthenium-Paraphenylene-Cyclopentadienyl Full-Sandwich Complexes: Cytotoxic Activity against A549 Lung Cancer Cell Line and DNA Binding Properties
Source: Molecules. 2023 Dec 19;29(1):17. doi: 10.3390/molecules29010017 (PMC10780011; doi:10.3390/molecules29010017)

checkCIF/PLATON report

Structure factors have been supplied for datablock(s) AG10_22_0m_a

THIS REPORT IS FOR GUIDANCE ONLY. IF USED AS PART OF A REVIEW PROCEDURE FOR PUBLICATION, IT SHOULD NOT REPLACE THE EXPERTISE OF AN EXPERIENCED CRYSTALLOGRAPHIC REFEREE.

No syntax errors found.   CIF dictionary   Interpreting this report

Datablock: AG10_22_0m_a

```
Bond precision:   C-C = 0.0105 A          Wavelength=0.71073

Cell:             a=10.5652(6)     b=13.4766(8)        c=10.7065(6)
                  alpha=90         beta=101.663(3)     gamma=90
Temperature:      296 K

                 Calculated                     Reported
Volume           1492.95(15)                    1492.95(15)
Space group      P 21/n                         P 21/n
Hall group       -P 2yn                         -P 2yn
Moiety formula   C28 H24 Ru2, 2(F6 P)           ?
Sum formula      C28 H24 F12 P2 Ru2             C28 H24 F12 P2 Ru2
Mr               852.55                         852.55
Dx,g cm-3        1.896                          1.897
Z                2                              2
Mu (mm-1)        1.213                          1.213
F000             836.0                          836.0
F000'            831.89
h,k,lmax         12,16,12                       12,16,12
Nref             2631                           2629
Tmin,Tmax        0.804,0.976                    0.667,0.746
Tmin'            0.785

Correction method= # Reported T Limits: Tmin=0.667 Tmax=0.746
AbsCorr = MULTI-SCAN

Data completeness= 0.999           Theta(max)= 24.998

R(reflections)= 0.0603( 1924)                            wR2(reflections)=
                                                         0.1151( 2629)
S = 1.069                    Npar= 291
```

The following ALERTS were generated. Each ALERT has the format
test-name_ALERT_alert-type_alert-level.
Click on the hyperlinks for more details of the test.

Alert level B

```
RINTA01_ALERT_3_B  The value of Rint is greater than 0.18
            Rint given   0.199
```

Author Response: : Crystals diffracted weakly. Attempts were made to grow larger and better diffracting crystals. Data was collected for three different crystals, with this one giving better results. Despite that the model is sound and agrees with other spectroscopic results.

Alert level C

```
PLAT088_ALERT_3_C Poor Data / Parameter Ratio ....................       9.03 Note
PLAT242_ALERT_2_C Low    'MainMol' Ueq as Compared to Neighbors of        Ru1 Check
PLAT260_ALERT_2_C Large Average Ueq of Residue Including        P1      0.119 Check
PLAT260_ALERT_2_C Large Average Ueq of Residue Including       P1'      0.108 Check
PLAT342_ALERT_3_C Low Bond Precision on  C-C Bonds ...............     0.0105 Ang.
PLAT906_ALERT_3_C Large K Value in the Analysis of Variance ......     17.053 Check
PLAT906_ALERT_3_C Large K Value in the Analysis of Variance ......      2.755 Check
```

Alert level G

```
PLAT003_ALERT_2_G Number of Uiso or Uij Restrained non-H Atoms ...         24 Report
PLAT020_ALERT_3_G The Value of Rint is Greater Than 0.12 .........      0.199 Report
PLAT083_ALERT_2_G SHELXL Second Parameter in WGHT  Unusually Large       5.06 Why ?
PLAT178_ALERT_4_G The CIF-Embedded .res File Contains SIMU Records          4 Report
PLAT187_ALERT_4_G The CIF-Embedded .res File Contains RIGU Records          2 Report
PLAT188_ALERT_3_G A Non-default SIMU Restraint Value has been used     0.0200 Report
PLAT188_ALERT_3_G A Non-default SIMU Restraint Value has been used     0.0200 Report
PLAT301_ALERT_3_G Main Residue  Disorder .............(Resd  1  )         33% Note
PLAT302_ALERT_4_G Anion/Solvent/Minor-Residue Disorder (Resd  2  )       100% Note
PLAT302_ALERT_4_G Anion/Solvent/Minor-Residue Disorder (Resd  3  )       100% Note
PLAT304_ALERT_4_G Non-Integer Number of Atoms in ..... (Resd  2  )       4.92 Check
PLAT304_ALERT_4_G Non-Integer Number of Atoms in ..... (Resd  3  )       2.08 Check
PLAT811_ALERT_5_G No ADDSYM Analysis: Too Many Excluded Atoms ....          ! Info
PLAT860_ALERT_3_G Number of Least-Squares Restraints .............        258 Note
PLAT883_ALERT_1_G No Info/Value for _atom_sites_solution_primary .     Please Do !
PLAT899_ALERT_4_G SHELXL2018 is Deprecated and Succeeded by SHELXL     2019/3 Note
PLAT909_ALERT_3_G Percentage of I>2sig(I) Data at Theta(Max) Still        69% Note
PLAT910_ALERT_3_G Missing # of FCF Reflection(s) Below Theta(Min).          3 Note
PLAT933_ALERT_2_G Number of HKL-OMIT Records in Embedded .res File          3 Note
PLAT961_ALERT_5_G Dataset Contains no Negative Intensities .......     Please Check
PLAT967_ALERT_5_G Note: Two-Theta Cutoff Value in Embedded .res ..       50.0 Degree
PLAT978_ALERT_2_G Number C-C Bonds with Positive Residual Density.          1 Info
```

0 **ALERT level A** = Most likely a serious problem - resolve or explain
1 **ALERT level B** = A potentially serious problem, consider carefully

```
 7 ALERT level C = Check. Ensure it is not caused by an omission or oversight
22 ALERT level G = General information/check it is not something unexpected

 1 ALERT type 1 CIF construction/syntax error, inconsistent or missing data
 7 ALERT type 2 Indicator that the structure model may be wrong or deficient
12 ALERT type 3 Indicator that the structure quality may be low
 7 ALERT type 4 Improvement, methodology, query or suggestion
 3 ALERT type 5 Informative message, check
```

It is advisable to attempt to resolve as many as possible of the alerts in all categories. Often the minor alerts point to easily fixed oversights, errors and omissions in your CIF or refinement strategy, so attention to these fine details can be worthwhile. In order to resolve some of the more serious problems it may be necessary to carry out additional measurements or structure refinements. However, the purpose of your study may justify the reported deviations and the more serious of these should normally be commented upon in the discussion or experimental section of a paper or in the "special_details" fields of the CIF. checkCIF was carefully designed to identify outliers and unusual parameters, but every test has its limitations and alerts that are not important in a particular case may appear. Conversely, the absence of alerts does not guarantee there are no aspects of the results needing attention. It is up to the individual to critically assess their own results and, if necessary, seek expert advice.

Publication of your CIF in IUCr journals

A basic structural check has been run on your CIF. These basic checks will be run on all CIFs submitted for publication in IUCr journals (*Acta Crystallographica*, *Journal of Applied Crystallography*, *Journal of Synchrotron Radiation*); however, if you intend to submit to *Acta Crystallographica Section C* or *E* or *IUCrData*, you should make sure that full publication checks are run on the final version of your CIF prior to submission.

Publication of your CIF in other journals

Please refer to the *Notes for Authors* of the relevant journal for any special instructions relating to CIF submission.

PLATON version of 06/07/2023; check.def file version of 30/06/2023

Datablock AG10_22_0m_a - ellipsoid plot

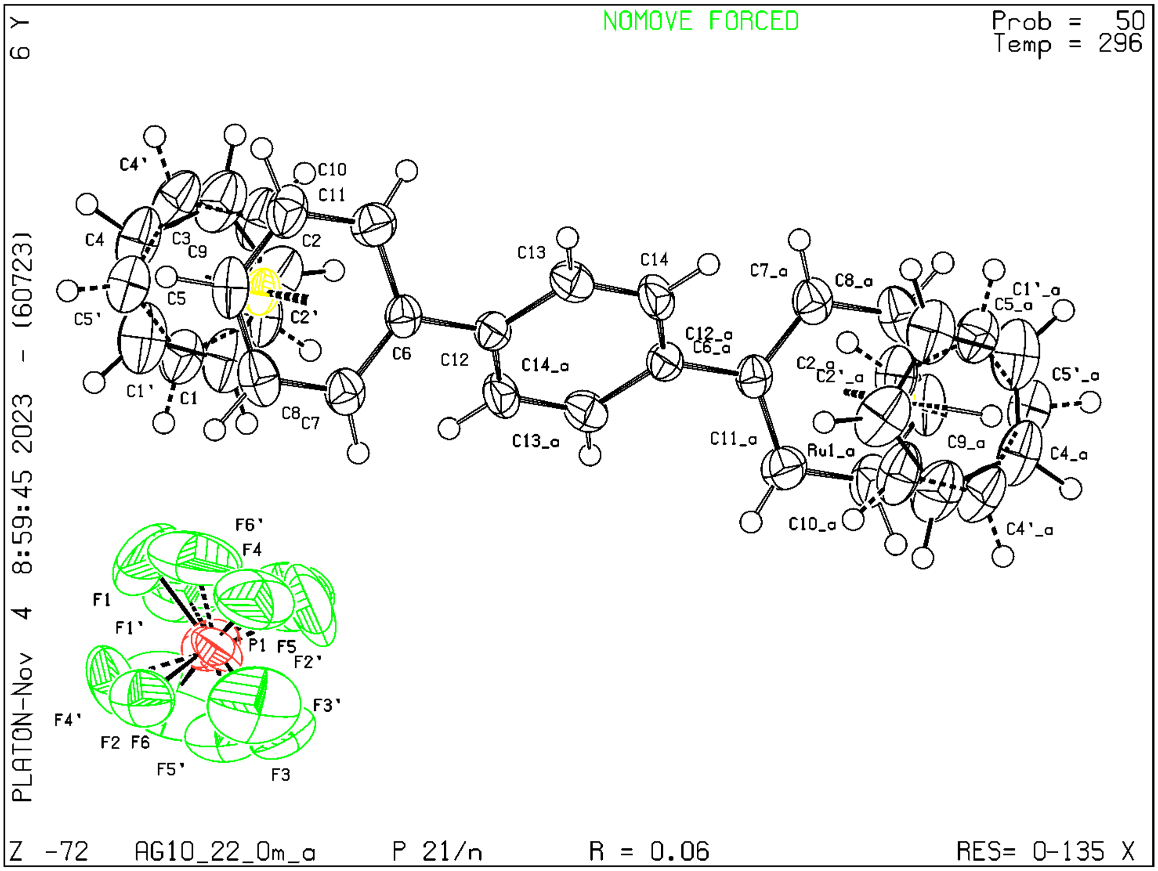

Supplement: Supplementary file 1 [file checkcif_4.pdf › checkcif_4.pdf]
